# Strategies for Navigating Magnetic Microrobots in Neurovascular Networks: A Numerical Analysis

**DOI:** 10.1002/smsc.202500180

**Published:** 2025-07-25

**Authors:** Pedro G. Alves, Maria Pinto, Rosa Moreira, Derick Sivakumaran, Fabian Landers, Maria Guix, Bradley J. Nelson, Andreas D. Flouris, Salvador Pané, Josep Puigmartí‐Luis, Tiago S. Mayor

**Affiliations:** ^1^ Transport Phenomena Research Centre (CEFT) Engineering Faculty Porto University Rua Dr. Roberto Frias 4200‐465 Porto Portugal; ^2^ Associate Laboratory in Chemical Engineering (ALICE) Engineering Faculty Porto University Rua Dr. Roberto Frias 4200‐465 Porto Portugal; ^3^ Experian 4200‐205 Porto Portugal; ^4^ Magnebotix Rütistrasse 14 8952 Zürich Schlieren Switzerland; ^5^ Multi‐Scale Robotics Lab ETH Zurich Tannenstrasse 3 8006 Zürich Switzerland; ^6^ Depart. de Ciència dels Materials i Química Física and Institut de Química Teòrica i Computacional University of Barcelona Martí i Franquès 1‐11 08028 Barcelona Spain; ^7^ FAME Laboratory Department of Exercise Science University of Thessaly Karies Trikala Thessaly 42100 Greece; ^8^ Institució Catalana de Recerca i Estudis Avançats (ICREA) Passeig Lluís Companys 23 08010 Barcelona Spain

**Keywords:** blood flow simulations, drug delivery, magnetic manipulation, microrobots, neurovascular networks, numerical simulations

## Abstract

First‐line therapy for ischemic stroke relies on the systemic administration of thrombolytics for dissolving clots affecting brain perfusion. However, because conservative dosages are used to avoid off‐target toxicity and side effects, the systemic route is often unable to deal with large clots in a timely manner. Targeted delivery of thrombolytics could be the solution if microrobots carrying the drugs could be navigated through the patient's neurovascular network to locally administer them directly to the clot. Herein, the steering of magnetic microrobots along a patient‐specific neurovascular network is numerically studied, for optimizing the navigation of the microrobots to target vessels often obstructed in ischemic stroke. It is found that spatially constant magnetic gradients can be used to navigate the microrobots to the target vessels and describe various navigation strategies that can be used by health professionals to reach different positions in the vasculature. Equations are developed to predict the required magnetic gradients as a function of the microrobot diameter, which are key for the development of magnetic navigation systems that can autonomously navigate microrobots through neurovascular networks. These findings open exciting possibilities for exploring targeted drug delivery approaches in clinical settings.

## Graphical abstract

Navigation of microrobots within vascular structures is key for targeted drug delivery. This work discusses strategies for navigating microrobots through a patient‐specific neurovascular network using magnetic gradients. These strategies and the equations developed for predicting the required magnetic gradients based on microrobot diameter are important for the development of magnetic navigation systems that can autonomously navigate microrobots through neurovascular networks.

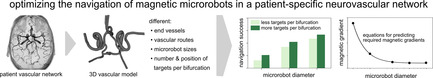

## 1 Introduction

Stroke is the second‐leading cause of death worldwide, affecting the elderly at higher rates.^[^ ^1^ ^]^ Of the global stroke incidence, two‐thirds are of ischemic type in which blood supply to an area of the brain is blocked by a clot, causing long‐term disability or even death.^[^ ^2^ ^]^ The first‐line therapy for ischemic strokes consists of the systemic administration of thrombolytics to dissolve the clot and restore blood flow.^[^ ^2^, ^3^, ^4^ ^]^ While being less invasive than thrombectomy (a procedure involving the use of a catheter to extract the clot), systemic administration is far from ideal, particularly in situations involving large clots that cannot be dissolved in a timely manner with the dosage of thrombolytics that avoid toxicity and side effects (e.g., bleeding due to the drug's blood thinning effect).^[^ ^5^ ^]^ The slow pace of systemic administration is particularly worrisome in ischemic stroke patients because of the pressing need to reperfuse the affected brain regions to avoid cognitive decline and loss of functionality. In all the above scenarios, a targeted delivery of thrombolytics could offer several advantages over the systemic approach.

Targeted delivery of thrombolytics could accelerate clot lysis and ensuing reperfusion because of the larger dosages of thrombolytics that could be locally delivered, without the side effects of a systemic approach. In this context, magnetic microrobots are promising candidates to serve as drug carriers in targeted drug delivery.^[^ ^6^, ^7^, ^8^ ^]^ On the one hand, their small size allows them to pass through narrow spaces and reach deep regions in the body with a minimally invasive procedure. On the other hand, their magnetic nature offers a way to externally control and steer them along complex vascular networks.^[^ ^9^, ^10^, ^11^, ^12^ ^]^ In other words, magnetic microrobots can be the ideal platform for transporting therapeutic agents^[^ ^7^ ^]^ and for enabling their localized delivery at specific vascular regions (e.g., arteries obstructed by clots). For this purpose, one needs to ensure that the microrobots can be navigated to the target vessels.

Both numerical and in vitro works have studied the use of magnetic fields and magnetic gradients to navigate microrobots in arteries and bifurcations. For instance, Bose and Banerjee^[^ ^13^ ^]^ calculated the 2D trajectory and capture of microrobots (0.25–4 μm) in a stenosed artery bifurcation to assess the effect of the flow Reynolds number, the microrobot size, and the magnetic field gradients, on the capture efficiency. They concluded that the use of higher magnetic field gradients, larger microrobots, and lower flow Reynolds number increases capture. Kenjereš and colleagues^[^ ^14^ ^]^ numerically studied the deposition of magnetic particles (0.25–4 μm) in the arteries of the brain and observed higher capture when using magnets in the arteries vicinity. Most in vitro experiments have studied how to use different locomotion strategies (i.e., tumbling, rolling, and propulsion)^[^ ^15^, ^16^ ^]^ and magnetic stimuli (i.e., oscillating fields, rotating fields, and field gradients)^[^ ^17^, ^18^, ^19^, ^20^, ^21^ ^]^ to navigate magnetic microrobots in phantom models of vascular systems.

In vivo works involving manipulation of magnetic objects in the arteries of animal models have also been conducted. For instance, Martel et al.^[^ ^22^ ^]^ used magnetic resonance imaging‐induced magnetic gradients to move a 1.5 mm bead along a bifurcation‐free portion of the carotid artery of a swine model. Also, Liu and colleagues^[^ ^23^ ^]^ manipulated a magnet in space to move an 800 μm microrobot within the femoral artery of a rodent. While the abovementioned works show that microrobots can be magnetically navigated in vascular structures, there is a lack of detailed quantitative information on the required magnetic gradients for different blood flow rates, vessel geometries, and microrobot sizes, for instance in the form of equations that can be implemented in algorithms controlling the operation of magnetic systems. To fill this gap, we recently studied the transport and navigation of microrobots along various cerebral bifurcations, to investigate the effect of microrobot size, artery geometry, blood velocity, microrobot entrance position, and target locations, on the magnetic gradients required to successfully navigate the microrobots toward a specific vessel.^[^ ^24^ ^]^ We observed that the microrobot size has the largest effect over the required magnetic gradients, and proposed correlations to predict the gradient magnitude based on the microrobot size. While these equations are important for guiding the development of magnetic manipulation systems and their navigation algorithms, it is nevertheless crucial to extend the above analysis to entire neurovascular networks, for studying the navigation of microrobots in conditions (i.e., geometries, blood flows) mimicking the final clinical application.

In this work, we study the transport and magnetic manipulation of microrobots through an entire neurovascular network, to identify the ideal magnetic gradients and strategies that can successfully navigate the microrobots toward vessels often obstructed in stroke patients. We numerically simulate the blood flow in a patient‐specific neurovascular network to analyze the effect of the bloodstream over the trajectories of microrobots of different sizes. We test and evaluate the success of three navigation strategies with different numbers and placements of intermediate targets, for navigating the microrobots toward the target vessels. We then develop equations for predicting the required magnetic gradients, as a function of the microrobot size. These results are important for the design and development of microrobots and magnetic navigation systems that can enable the translation of targeted drug delivery approaches to clinical settings.

## 2 Experimental Section

### 2.1 Problem Formulation

The transport of microrobots through a patient‐specific neurovascular network was numerically simulated to study how magnetic manipulation could be used for navigating the microrobots to specific target vessels where clots are often found in ischemic stroke. With the goal of identifying the ideal magnetic gradients and navigation strategy, we considered that the microrobots would be transported by the bloodstream and that magnetic gradients would be used to steer the microrobots along various routes (see Section 2.2.2 for details) through the neurovascular network, from the inlet to the target vessels. To navigate microrobots with different sizes along the mentioned routes, we considered the existence of intermediate targets placed at various distances around the bifurcations or along the entire path until the target vessel. We then varied the number and position of the intermediate targets to investigate their effect on the ability to navigate the microrobots along each route and on the magnetic gradients that are required in each scenario. In these analyses, the microrobot motion was assumed to be affected by drag, gravitational and buoyancy forces, as well as the magnetic force imposed by the magnetic gradients. Finally, to characterize the ability to navigate microrobots along the neurovascular network, we calculated the navigation success defined as the ratio between the number of microrobots reaching the target vessels and the total number of microrobots entering the vascular network. Based on this definition, the navigation success ranges from 0% (no microrobot reaching the target vessels) to 100% (all microrobots reaching the target vessels).

### 2.2 Modeling Assumptions and Boundary Conditions

#### 2.2.1 Neurovascular Network

**Figure 1:**
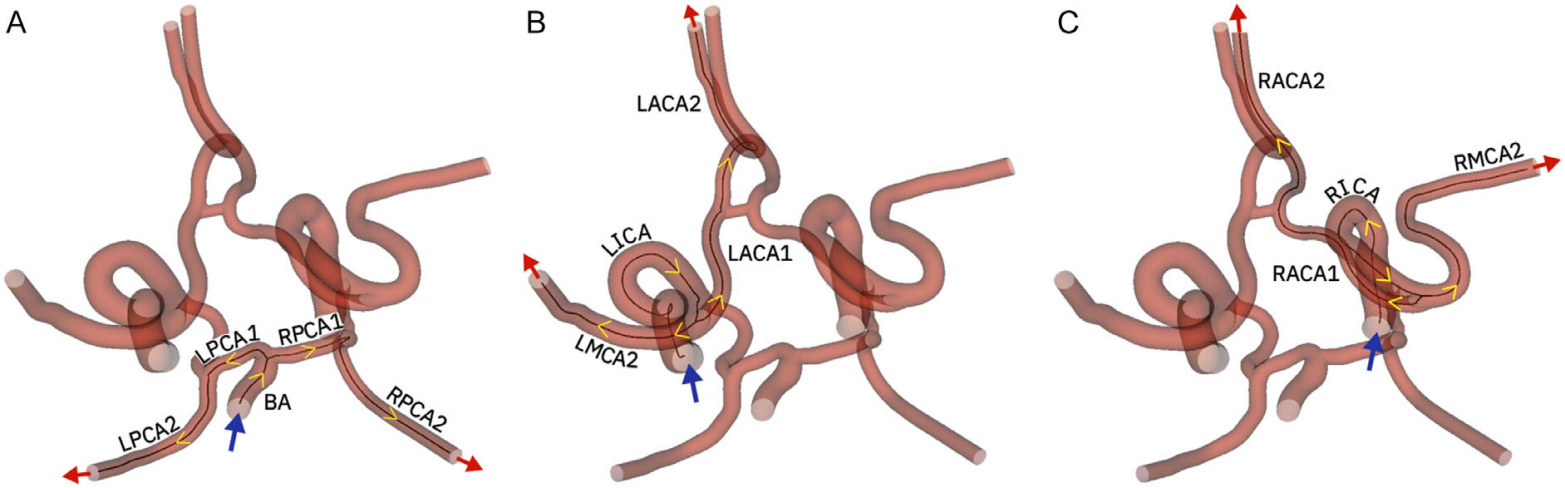
3D model of the neurovascular network used for the simulations. It includes the basilar artery (BA), the left and right internal carotid arteries (LICA and RICA, respectively), and two segments of the left and right posterior, anterior, and middle cerebral arteries (LPCA, RPCA, LMCA, RMCA, LACA, and RACA, respectively). Blood enters through the BA, LICA, and RICA (represented by the blue arrows) and exits through the second segment of each major artery (LPCA2, RPCA2, LMCA2, RMCA2, LACA2, and RACA2, red arrows). For simplicity, the LMCA1 and RMCA1 captions are not shown. The six routes along which the microrobots are navigated are represented by the dark centerlines through the relevant arteries, and the direction that the microrobots follow is represented by the yellow arrows: A) Two possible routes connecting the BA and the LPCA2 or the RPCA2. B) Two possible routes connecting the LICA and the LMCA2 or the LACA2. C) Two possible routes connecting the RICA and the RMCA2 or the RACA2.

A 3D model of the main arteries of the neurovascular network, obtained from medical images of a healthy patient,^[^ ^25^ ^]^ was used to numerically simulate the blood flow and the microrobots motion and trajectory. Featuring the Circle of Willis and the main branching arteries (**Figure** **1**), the model includes the basilar artery (BA), the left and right internal carotid arteries (LICA and RICA, respectively), and two segments of the left and right posterior, anterior, and middle cerebral arteries (LPCA, RPCA, LMCA, RMCA, LACA, and RACA, respectively). Blood enters through the BA, LICA, and RICA (represented by the blue arrows in Figure 1) and exits through the second segment of every main artery (i.e., LPCA, RPCA, LMCA, RMCA, LACA, and RACA; red arrows in Figure 1). The average diameter (*D*_a_) and centerline length (*L*) of each relevant artery segment are shown in **Table** **1**.

**Table 1:** Average diameter and centerline length of each relevant artery in the 3D neurovascular model.

Artery names		Average diameter, *D*_a_ [mm]	Centerline length, *L* [mm]
Basilar	BA	3.10	11.47
Left internal carotid	LICA	4.04	47.16
Right internal carotid	RICA	4.06	40.73
1st segment left posterior	LPCA1	1.94	10.04
2nd segment left posterior	LPCA2	2.09	24.57
1st segment right posterior	RPCA1	2.09	13.18
2nd segment right posterior	RPCA2	2.10	30.06
1st segment left middle	LMCA1	3.42	6.66
2nd segment left middle	LMCA2	2.83	31.80
1st segment right middle	RMCA1	3.09	7.22
2nd segment right middle	RMCA2	2.73	41.30
1st segment left anterior	LACA1	2.37	20.89
2nd segment left anterior	LACA2	2.05	38.18
1st segment right anterior	RACA1	1.90	28.98
2nd segment right anterior	RACA2	1.90	36.28

#### 2.2.2 Navigation Routes and Intermediate Targets

**Figure 2:**
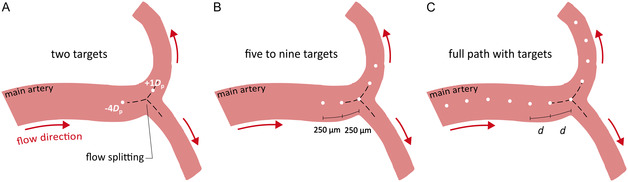
Navigation strategies considered in this work, featuring different number and position of the intermediate targets (white dots) relative to the point of flow splitting (intersection of the dashed lines), along with the direction of the blood flow (red arrows): A) Two‐target strategy featuring two intermediate targets at each bifurcation, one upstream and one downstream the point of flow splitting. In the illustration, the targets are positioned at a distance from the point of flow splitting, expressed in microrobot diameters (i.e., −4*D*_p_ and +1*D*_p_ refer to four diameters upstream and one diameter downstream the point of flow splitting, respectively). B) Five‐to‐nine‐target strategy featuring five to nine targets at each bifurcation, two to four upstream and downstream the point of flow splitting, and one at the point of flow splitting. In the illustration, the targets are placed 250 μm apart, but intertarget distances of 500 and 1000 μm were also considered in this work. C) Full path with targets strategy featuring targets distributed along the entire path, considering five different intertarget distances defined as multiples of the radial distance available for the larger microrobots to move inside each artery without touching the walls (i.e., *d* = *D*_a_–1000 μm, where *D*_a_ is the artery diameter, Table 1). We considered intertarget distances of 1*d*, 2*d*, 3*d*, 4*d*, and 5*d*.

**Figure 3:**
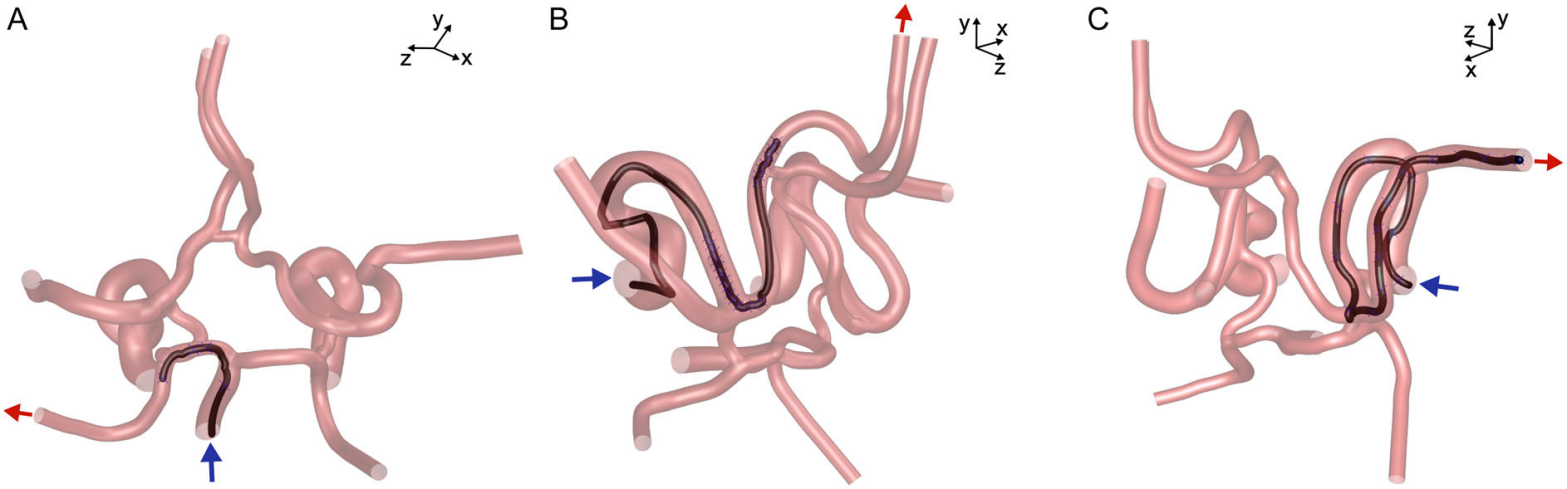
Examples of successful navigation of three microrobots (1000 μm) through three different routes using each of the three navigation strategies, for which the magnetic gradient is calculated at every time step. The inlet and outlet associated with each route are represented by the blue and red arrows, respectively. The microrobots are represented by their spherical volume throughout their trajectory and the intermediate targets are represented by the blue crosses. A) Navigation of a microrobot entering the BA and steered to the LPCA outlet using the two‐target strategy, with targets placed four microrobots diameter upstream and downstream the position of flow splitting in each bifurcation. B) Navigation of a microrobot entering the LICA and steered to the LACA using the five‐to‐nine‐target strategy with using seven targets, 1000 μm apart in each bifurcation. C) Navigation of a microrobot entering the RICA and steered to the RMCA using the full path with target strategy with the targets separated by five times the radial distance available for the 1000 μm microrobot to move inside each artery without touching the walls (i.e., 5 × (*D*_*a*_−1000 μm) in Table 2). In each of the images above, the microrobot trajectory is assumed to end when the microrobot reaches the last target.

Literature studies related to ischemic stroke indicate that clots often obstruct the blood flow in the posterior, middle, and anterior cerebral arteries.^[^ ^26^, ^27^, ^28^, ^29^ ^]^ For that reason, we investigated the navigation of microrobots to these regions. We considered a total of six different routes with the shortest path between the microrobots entrance position and the target vessels: 1) two routes connecting the basilar artery (BA), with the left/right posterior cerebral arteries (LPCA2/RPCA2; Figure 1A), 2) two routes connecting the left internal carotid artery (LICA) with the left middle and anterior cerebral arteries (LMCA2/ LACA2, Figure 1B), and 3) two routes connecting the right internal carotid artery (RICA) with the right middle and anterior cerebral arteries (RMCA2/RACA2, Figure 1C). Each route includes two to three different bifurcations that the microrobots must navigate to reach the target vessels. To ensure that the microrobots follow the predetermined route, we considered the existence of intermediate targets placed at different positions along the arteries (**Figure** **2**).

In a previous work on the navigation of magnetic particles along bifurcations,^[^ ^24^ ^]^ we observed that the magnetic gradient required to ensure a successful navigation of the microrobots along a given path varied with the positioning of the intermediate targets. Building on this, we considered three different navigation strategies (Figure 2) with different numbers and positions of the intermediate targets, to investigate how these affect the ability to navigate the microrobots along the six routes mentioned earlier. Specifically, we considered navigation strategies featuring: 1) two targets at each bifurcation, one upstream and another downstream the point of flow splitting (Figure 2A); 2) five to nine targets at each bifurcation, two to four upstream and downstream the point of flow splitting, and one target at the point of flow splitting (Figure 2B); and 3) full path with targets (Figure 2C).

For each of the three navigation strategies, we considered different positions of the targets (**Table** **2**), to identify the intertarget distance that maximizes the number of microrobots reaching the desired vessel (where the clot is located). For the strategy with two targets (Figure 2A), we considered four positions upstream and downstream the flow splitting, defined in relation to the microrobot diameter (i.e., −4*D*_*p*_, −3*D*_*p*_, −2*D*_*p*_, and −1*D*_*p*_, for the upstream positions, and +1*D*_*p*_, +2*D*_*p*_, +3*D*_*p*_, and +4*D*_*p*_, for the downstream positions, Table 2), for a total of 16 possible combinations. For the strategy with five to nine targets (Figure 2B), we considered two, three, or four targets upstream and downstream the point of flow splitting, and three different intertarget distances (i.e., 250, 500, and 1000 μm, Table 2), for a total of nine combinations. Finally, for the strategy with the full path covered with targets (Figure 2C), we considered five different intertarget distances (*d*) calculated as multiples of the radial distance available for the larger microrobot to move inside each artery without touching the walls. Given the size of the arteries and the size of the larger microrobots (*D*_*p*_ = 1000 μm, Section 2.2.4), we considered intertarget distances of 1*d*, 2*d*, 3*d*, 4*d*, and 5*d* (Table 2), with *d* calculated as *D*_*a*_ – 1000 μm, where *D*_*a*_ is the artery diameter (Table 1).

**Table 2:** Variations considered for each of the three navigation strategies. For the strategy with two targets, four positions upstream/downstream were considered expressed in relation to the microrobot diameter (i.e., −1*D*_p_ refers to one microrobot diameter upstream the point of flow splitting and +1*D*_p_ refers to one diameter downstream the same point), for a total of 16 combinations. In the five‐to‐nine‐target strategy, three variations in the number of targets (i.e., five, seven, or nine) and three variations in the intertarget distance (i.e., 250, 500, or 1000 μm) were considered for a total of nine combinations. Finally, in the strategy with the full path covered with targets, five different intertarget distances (*d*) were considered, from *d* to 5*d*, with *d = D*_*a*_ *–*1000 μm, where *D*_*a*_ corresponds to the artery diameter (Table 1).

Two targets	Five to nine targets	Full path with targets
–1*D*_p_ + 1*D*_p_	5 targets, 250 μm apart	1 × (*D*_*a*_−1000 μm) apart
–1*D*_p_ + 2*D*_p_	7 targets, 250 μm apart	2 × (*D*_*a*_−1000 μm) apart
–1*D*_p_ + 3*D*_p_	9 targets, 250 μm apart	3 × (*D*_*a*_−1000 μm) apart
–1*D*_p_ + 4*D*_p_	5 targets, 500 μm apart	4 × (*D*_*a*_−1000 μm) apart
–2*D*_p_ + 1*D*_p_	7 targets, 500 μm apart	5 × (*D*_*a*_−1000 μm) apart
–2*D*_p_ + 2*D*_p_	9 targets, 500 μm apart	–
–2*D*_p_ + 3*D*_p_	5 targets, 1000 μm apart	–
–2*D*_p_ + 4*D*_p_	7 targets, 1000 μm apart	–
–3*D*_p_ + 1*D*_p_	9 targets, 1000 μm apart	–
–3*D*_p_ + 2*D*_p_	–	–
–3*D*_p_ + 3*D*_p_	–	–
–3*D*_p_ + 4*D*_p_	–	–
–4*D*_p_ + 1*D*_p_	–	–
–4*D*_p_ + 2*D*_p_	–	–
–4*D*_p_ + 3*D*_p_	–	–
–4*D*_p_ + 4*D*_p_	–	–

#### 2.2.3 Blood Flow

The blood was treated as a non‐Newtonian, incompressible fluid with a density of 1060 kg m^−3^ and an apparent viscosity described by the Carreau model.^[^ ^30^, ^31^ ^]^ The Carreau model was chosen due to its good agreement with experimental data^[^ ^32^ ^]^ for both low and high shear rates:

(1) $$\eta =\eta_{\infty }+\left(\eta_{0}-\eta_{\infty }\right){\left[1+{\left(\lambda \dot{\gamma }\right)}^{2}\right]}^{\frac{n-1}{2}}$$

where $\dot{\gamma }$ stands for the shear rate, $\eta_{0}$ = 0.056 Pa s and $\eta_{\infty }$ = 0.00345 Pa s are the limits of the apparent viscosity for low and high shear rates, respectively, and *λ* = 3.313 s and *n* = 0.3568 are fitting parameters.^[^ ^33^ ^]^

The blood flows in the neurovascular network in the laminar regime given the associated Reynolds number (i.e., Re < 2000).^[^ ^34^ ^]^ We considered the blood flow as nonpulsatile because steady flow conditions can accurately capture the blood flow distribution in the neurovascular network^[^ ^35^, ^36^, ^37^ ^]^ and predict relevant hemodynamic parameters (i.e., flow profile and distribution) with lower computational cost compared to transient approaches (more details in Supplementary Information).^[^ ^38^, ^39^, ^40^, ^41^, ^42^ ^]^

The total cerebral blood flow rate considered in this work (*Q*_*T*_ = 12 mL s^−1^) was obtained by averaging the values found in various in vivo measurements reported in the literature,^[^ ^43^, ^44^, ^45^, ^46^ ^]^ covering a total of 232 patients. The total cerebral blood flow rate was distributed to the various inlets (i.e., BA, LICA, and RICA; Figure 1) based on the average of the flow rate ratios reported for each artery in the aforementioned works (**Table** **3**). Similarly, the total blood flow rate was distributed to each of the outlets (i.e., PCAs, MCAs, and ACAs; Figure 1), based on the average of the reported flow rate ratios for each artery (Table 3).^[^ ^47^, ^48^, ^49^, ^50^, ^51^ ^]^ The blood flow rates at each of the inlets and outlets were calculated by multiplying the total blood flow rate by the respective average flow rate ratio (Table 3). Using these flow rates, we ran an initial simulation to calculate the pressures at the inlets and outlets ensuring the mentioned flow rate distribution and then imposed the obtained pressures (after scaling to match physiological pressures) as boundary conditions in the subsequent simulations.

**Table 3:** Flow rate ratios attributed to each of the inlets and outlets of the 3D network model, as well as the corresponding blood flow rate imposed as boundary condition. The total blood flow rate and the flow rate ratios considered for each artery were estimated as the average of various values reported in the literature,^[^ ^43^, ^44^, ^45^, ^46^, ^47^, ^48^, ^49^, ^50^, ^51^ ^]^ covering a total of 232 patients.

Inlets/outlets	Flow rate ratio [%]	Imposed blood flow rate, *Q*_*T*_ [mL s^−1^]
BA	20.47	2.46
LICA	40.43	4.85
RICA	39.10	4.69
LPCA	13.52	1.62
RPCA	13.38	1.60
LMCA	24.76	2.97
RMCA	26.21	3.15
LACA	11.05	1.33
RACA	11.08	1.33

In the 3D model of the neurovascular network, each of the three inlets (i.e., BA, LICA, and RICA) were extruded “backwards” (length of *L*_e_ ≈ 0.06 Re·*D*_a_, Figure S2A, Supporting Information) to ensure that the blood flow entering the neurovascular network was fully developed,^[^ ^30^ ^]^ as that is a better representation of the blood flow reaching the brain rather than having a homogenous/flat flow profile imposed as boundary condition (Table 3). No‐slip condition was assumed at the walls of the arteries.

#### 2.2.4 Microrobots and their Interaction with the Blood and the Vessel Walls

The motion of the microrobots through the neurovascular network was calculated based on a Lagrangian approach, where each microrobot is represented by a point mass that is tracked in time and space, for predicting its trajectory. The microrobots were modeled as smooth spheres for two reasons: 1) the hydrodynamics of spheres have been extensively studied experimentally and the drag coefficient for spherical objects can be accurately predicted based on the corresponding Reynolds number, and 2) the correlations for predicting the drag coefficient for spheres do not change with their orientation because they have rotational symmetry. For these reasons, the trajectory of spherical microrobots is easier to predict than that of more complex shapes such as rods or helices. The microrobot interaction with the blood flow was modeled via a one‐way approach, in which the blood flow is assumed to affect the motion of the microrobots via the induced drag force, and the microrobots volume is assumed not to affect the blood flow. We used a one‐way approach, in line with various works in the literature,^[^ ^14^, ^18^, ^52^, ^53^, ^54^ ^]^ to enable the simulation of many different scenarios in a timely manner, given its lower computational cost and interesting accuracy in the prediction of an object trajectory.

In a standard one‐way approach, microrobots are treated as points rather than volumes, which can allow for nonphysical situations where part of the microrobot volume falls outside the arteries’ walls. To avoid this, in this work, we prevent the microrobot centers from being less than one radius away from the artery walls, at which point an impact with the wall occurs. This implies that the microrobots can, at most, touch the artery walls but not cross them, which provides a more realistic prediction of the trajectories and collisions occurring as the microrobots move through the neurovascular network.

To model the collision of the microrobots with the walls and the resulting effects over their velocity, we used the coefficient of restitution (COR), a parameter describing the degree of elasticity of collisions. In fully elastic collisions (COR = 1), microrobots retain all the momentum, whereas in fully inelastic collisions (COR = 0), microrobots would lose all the momentum and attach to the wall. Various numerical works involving collisions of particles with vessel walls^[^ ^53^, ^55^, ^56^, ^57^, ^58^, ^59^ ^]^ use very different coefficients of restitution (i.e., 0.25–1.0), therefore not being clear what value best describes the collision of microrobots in neurovascular networks. For this reason, to minimize the effect of the coefficient of restitution over the predicted microrobot trajectories, we considered the collisions to be fully elastic (COR = 1), as inelastic collisions (COR < 1) would reduce the microrobots momentum and lead to their accumulation near the walls, two effects that would delay the microrobots progression along the neurovascular network and increase the calculation time (see Table S2 in the Supplementary Information).

We assumed that the entire volume of the microrobots (*V*_p_) consists of magnetic material only (i.e., iron oxide), with a density of 5200 kg m^−3^,^[^ ^60^ ^]^ that has reached its saturation magnetization (*M*_S_) of 5 × 10^5^ A m^−1^.^[^ ^61^, ^62^ ^]^ We considered the saturated state so that any given magnetic force (${\vec{F}}_{Magnetic}$) can be imposed using the lowest possible magnetic field gradient ($\nabla \vec{B})$, as described by

(2) $${\vec{F}}_{Magnetic}=V_{p}\cdot M_{s}\cdot \nabla \vec{B}\;\;\Leftrightarrow \;\;\nabla \vec{B}=\frac{{\vec{F}}_{Magnetic}}{V_{p}\cdot M_{s}}$$

The microrobots were assumed to have diameters (*D*_*p*_) of 50, 100, 250, 500, and 1000 μm, to study a broad range of sizes requiring very different magnetic gradients for navigation (Equation 2). We considered larger microrobots than those typically reported in the literature about magnetic navigation (where typically *D*_*p*_ ≪ 50 μm),^[^ ^13^, ^14^, ^54^, ^62^, ^63^ ^]^ as they have higher drug‐loading capacity, something important for drug delivery therapies.^[^ ^64^, ^65^, ^66^ ^]^ The microrobots were assumed to enter the neurovascular network through each of the three inlets (i.e., BA, LICA and RICA; Figure 1 and S2A, Supporting Information) and be transported by the blood flow, thus having the same velocity as the blood at their entrance positions (Figure S2B and S2C, Supporting Information).

### 2.3 Numerical Methods

A computational fluid dynamics approach based on the finite‐volume method (FVM)^[^ ^67^, ^68^ ^]^ was used for the calculation of the blood flow in the 3D model of the neurovascular network. The blood pressure and velocity were calculated by coupling the continuity and the Navier–Stokes equations for an incompressible non‐Newtonian fluid, given respectively by

(3) $$\nabla {\vec{u}}_{f}=0$$

(4) $$\rho_{f}\frac{\partial {\vec{u}}_{f}}{\partial t}=-\nabla p+\eta \nabla^{2}{\vec{u}}_{f}+\rho_{f}\vec{g}$$

where ${\vec{u}}_{f}$ is the flow velocity, $\rho_{f}$ is the fluid density, p is pressure, ∇ and $\nabla^{2}$ are the divergence and Laplacian operators, and $\vec{g}$ is the gravitational acceleration. A steady‐state, double‐precision, pressure‐based solver was used with second‐order discretization and velocity‐pressure coupling. Simulations were done using an unstructured mesh that can more accurately discretize the complex geometry of the uneven and tortuous arteries of the neurovascular network (Figure 1). Mesh‐independent results with low computational cost were achieved for a mesh with ≈6 million cells with maximum mesh element size of 0.175 mm (Figure S1A and S2C, Supporting Information). A convergence criterion of 10^−4^ was considered for the continuity and velocity was found to be adequate, with stricter criteria producing similar results (Figure S1B, Supporting Information).

The calculation of the microrobot velocity and position was done by integrating the force balance equation of the forces acting on the microrobot over discrete time steps (Equation 5). When injected into the bloodstream, a microrobot may be subjected to multiple forces, as a result of drag, buoyancy, gravity, pressure gradient, virtual mass, Brownian motion, and Saffmann's lift. Yet not all are relevant for navigation through the neurovascular network. The pressure gradient and virtual mass forces are significant only when $\rho_{f}/\rho_{p}$ ≈ 1 (in this work $\rho_{f}/\rho_{p}$ ≈ 0.2),^[^ ^54^ ^]^ and the Brownian motion and Saffmann's lift forces are relevant only for sub‐micron microrobots (in this work *D*_p_ > 50 μm).^[^ ^69^ ^]^ Thus, only the drag, buoyancy, and gravity forces, as well as the external magnetic force used for navigation, are assumed to contribute to the total (net) force ${\vec{F}}_{T}$ ^[^ ^70^ ^]^

(5) $$\underset{{\vec{F}}_{T}}{\underbrace{m_{p}\frac{d{\vec{u}}_{p}}{dt}}}=\underset{{\vec{F}}_{Drag}}{\underbrace{m_{p}\frac{{\vec{u}}_{f}-{\vec{u}}_{p}}{\tau_{p}}}}+\underset{{\vec{F}}_{Gravity}-{\vec{F}}_{Buoyancy}}{\underbrace{m_{p}\frac{\vec{g}\left(\rho_{p}-\rho_{f}\right)}{\rho_{p}}}}+\underset{{\vec{F}}_{Magnetic}}{\underbrace{V_{p}\cdot \nabla \vec{B}\cdot M_{s}}}$$

where $m_{p}$ is the microrobot mass, ${\vec{u}}_{p}$ is the microrobot velocity, *t* is time, $\tau_{p}$ is the microrobot relaxation time, and $\rho_{p}$ is the microrobot density. Moreover, $V_{p}$ is the microrobot volume, $\nabla \vec{B}$ is the magnetic gradient, and $M_{s}$ is the saturation magnetization. Using Equation 5, we compute the net force acting on the microrobot when moving along the neurovascular network under the effect of a magnetic field gradient. The microrobot velocity is then obtained by analytical integration of Equation 5 with respect to time (*t*)

(6) $${\vec{u}}_{p}^{i+1}={\vec{u}}_{f}^{i}+e^{-\frac{\Delta t}{\tau_{p}}}\left({\vec{u}}_{p}^{i}-{\vec{u}}_{f}^{i}\right)-\tau_{p}\left(e^{-\frac{\Delta t}{\tau_{p}}}-1\right)\left[\frac{\vec{g}\left(\rho_{p}-\rho_{f}\right)}{\rho_{p}}+\frac{V_{p}\cdot \nabla \vec{B}\cdot M_{s}}{m_{p}}\right]$$

where *i* is the current iteration of the calculation. The microrobot position can be obtained by replacing the velocity ${\vec{u}}_{p}$ in Equation 6 by its derivative form ${\vec{u}}_{p}=\frac{d{\vec{x}}_{p}}{dt}$, followed by its analytical integration with respect to time

(7) $${\vec{x}}_{p}^{i+1}={\vec{x}}_{p}^{i}+\Delta t\left[{\vec{u}}_{f}^{i}+\tau_{p}\left[\frac{\vec{g}(\rho_{p}-\rho_{f})}{\rho_{p}}+\frac{V_{p}\cdot \nabla \vec{B}\cdot M_{s}}{m_{p}}\right]\right]+\tau_{p}\left(1-e^{-\frac{\text{Δ}t}{\tau_{p}}}\right)\left[{\vec{u}}_{p}^{i}-{\vec{u}}_{f}^{i}-\tau_{p}\left[\frac{\vec{g}(\rho_{p}-\rho_{f})}{\rho_{p}}+\frac{V_{p}\cdot \nabla \vec{B}\cdot M_{s}}{m_{p}}\right]\right]$$

The microrobot trajectory is then calculated by iterating between Equations 6 and 7 for a time step (Δt) of $10^{-5}\cdot {\left(u_{p}^{i}+u_{f}^{i}\right)}^{-1}$. The value 10^−5^ [s] was found to generate microrobot trajectories that are independent of the chosen time step (i.e., smaller time steps yield similar trajectories). At every time step, the magnetic gradient that moves the microrobot from its position toward the next intermediate target is obtained by isolating $\nabla \vec{B}$ in Equation 7

(8) $$\nabla \vec{B}=\frac{\vec{d}-{\vec{u}}_{f}\cdot t-{\vec{u}}_{p}\cdot \tau_{p}+{\vec{u}}_{f}\cdot \tau_{p}+\tau_{p}\cdot e^{-\frac{t}{\tau_{p}}}\cdot \left({\vec{u}}_{p}-{\vec{u}}_{f}\right)+\tau_{p}\cdot \frac{\vec{g}\left(\rho_{p}-\rho_{f}\right)}{\rho_{p}}\cdot \left(\tau_{p}-t-\tau_{p}\cdot e^{-\frac{t}{\tau_{p}}}\right)}{\frac{V_{p}\cdot M_{s}}{m_{p}}\cdot \tau_{p}\cdot \left(t-\tau_{p}+\tau_{p}\cdot e^{-\frac{t}{\tau_{p}}}\right)}$$

where $\vec{d}={\vec{x}}_{p}^{i+1}-{\vec{x}}_{p}^{i}$ is the distance to the next target, and $t=∥\vec{d}∥\cdot ∥{\vec{u}}_{i}^{f}{∥}^{-1}$ is the time that the microrobot would take to reach the next target if moving with the velocity of the blood flow at the current position.

At every iteration over time, the microrobot trajectory and motion are obtained by first computing with Equation 8 the magnetic gradient that moves the microrobot from its position toward the next target, which is then used in Equation 6 and 7 to calculate the microrobot velocity and position, respectively. The iterative calculation ends when the microrobot reaches the last target or an outlet.

### 2.4 Validation

The flow predicted using the described numerical approach was validated by comparing the flow profile at an inlet with the analytical solution for a fully developed laminar flow in a pipe (Figure S1D, Supporting Information). The strong agreement of the predicted flow profile with the analytical solution shows that the flow is fully developed and accurately predicted. This indicates that the described numerical approach can predict the flow in vascular structures.

The validation of the magnetic navigation of the microrobots described by Equation 5 was done via two different comparisons. First, the numerical work of Haverkort et al.^[^ ^71^ ^]^ was replicated using the modeling approach described in the previous sections, and the numerical results obtained were compared with those in the mentioned work. Specifically, microrobots of different sizes (0.05 to 2 μm) were injected into a 90° bended tube with blood flowing at 0.10 m s^−1^ while a magnetic field generated by a wire placed nearby was used to capture the microrobots. The capture efficiency obtained using our modeling approach closely follows the efficiency reported in the mentioned work (Figure S1E, Supporting Information), which shows that our numerical approach can be used to predict the effect of the magnetic force on the trajectory of microrobots transported in blood flows.

For the second comparison, we first ran a series of in vitro experiments in a Y‐bifurcation, considering 32 different experimental conditions involving changes in magnetic gradients and flow velocity. A 1.4 mm diameter ferromagnetic sphere was injected into the bifurcation with flowing water, and an external magnetic field gradient was used to steer the sphere into the desired outlet. Eight magnetic gradient magnitudes in the range 0–700 mT m^−1^ and four flow velocities in the range 0.10–0.40 m s^−1^ were combined in a systematic way to generate 32 independent experimental conditions that were repeated 20 times for a total of 640 experiments (i.e., 8 magnetic gradient values × 4 flow velocities × 20 repetitions). The navigation success was calculated as the ratio between the number of spheres reaching the desired outlet and the total number of spheres entering the bifurcation (20 spheres). We then prepared a 3D model of the bifurcation and numerically replicated the experiments by iterating Equation (6), (7) for predicting the sphere motion and trajectory along the bifurcation. The magnetic field gradients imposed during the in vitro experiments were used at each iteration to calculate the resulting magnetic force acting on the spheres. The navigation success was then calculated in the same manner as the in vitro experiments. The results (Figure S10, Supporting Information) show that the developed numerical approach allows to predict the magnetic force and its effect on the trajectory of spheres navigated through bifurcations. For this reason, it can also be used to predict the motion of microrobots along the various bifurcations of the neurovascular network considered in this work. For more details on the in vitro experiments, see ref. [24].

### 2.5 Cases Considered for the Numerical Simulations

The motion and magnetic navigation of the microrobots in the neurovascular network were simulated considering the various possibilities of navigation strategy (i.e., the three navigation strategies and their variations, Table 2). The performance of each strategy in steering the microrobots along the six routes (Figure 1) leading to regions in the brain often affected by strokes^[^ ^26^, ^27^, ^28^, ^29^ ^]^ was assessed by calculating the navigation success defined as the ratio between the number of microrobots successfully reaching the target vessels and the total number of microrobots released at the inlets of the neurovascular network. We considered five microrobot diameters (50–1000 μm), each requiring necessarily different magnetic gradients to be navigated, with sizes relevant for drug delivery therapies.^[^ ^64^, ^65^, ^66^ ^]^ As detailed in Section 2.2.2, we used three different navigation strategies based on the number and positioning of the intermediate targets (i.e., two targets per bifurcation [16 cases], five to nine targets per bifurcation [9 cases], and the full path with targets [5 cases]; Figure 2), amounting to 16 + 9 + 5 = 30 different cases (Table 2). In an initial approach, we computed the magnetic gradients at every time increment (Equation 8) along the trajectory of microrobots released from 16 different representative positions at each inlet (Figure S2B, Supporting Information), for a total of 14400 simulation scenarios (i.e., 6 cerebral routes × 5 microrobot diameters × 30 navigation cases × 16 entrance positions).

### 2.6 Statistical Analysis

The magnetic gradient data was obtained at each microrobot time step for *n* = 14400 independent simulations (the microrobot time step varies with the microrobot and flow velocities, see Section 2.3). The average magnetic gradient in the G1 and G2 regions was calculated considering all the magnetic gradients in those regions, for every bifurcation that was successfully navigated in each simulation scenario. The average magnetic gradients were then grouped by microrobot size (generating groups with *n* = 2880 magnetic gradients), and the median magnetic gradient of each group was calculated. The least squares method was used to find a second‐order, linear fitting equation of the median magnetic gradients as a function of the microrobot size. Statistical significance was set at *p* < 0.05 and all calculations were carried out using MATLAB.

## 3 Results and Discussion

### 3.1 Navigation in the Neurovascular Network

The navigation success along the six routes was analyzed for each microrobot diameter and manipulation strategy, with the magnetic gradient being updated at every time step as the microrobots moved along the neurovascular network. Examples of successful navigation through three of the six routes (i.e., BA to LPCA, LICA to LACA, and RICA to RACA) are shown in **Figure** **3**, for a microrobot with a diameter of 1000 μm, represented along each trajectory by its volume.

**Figure 4:**
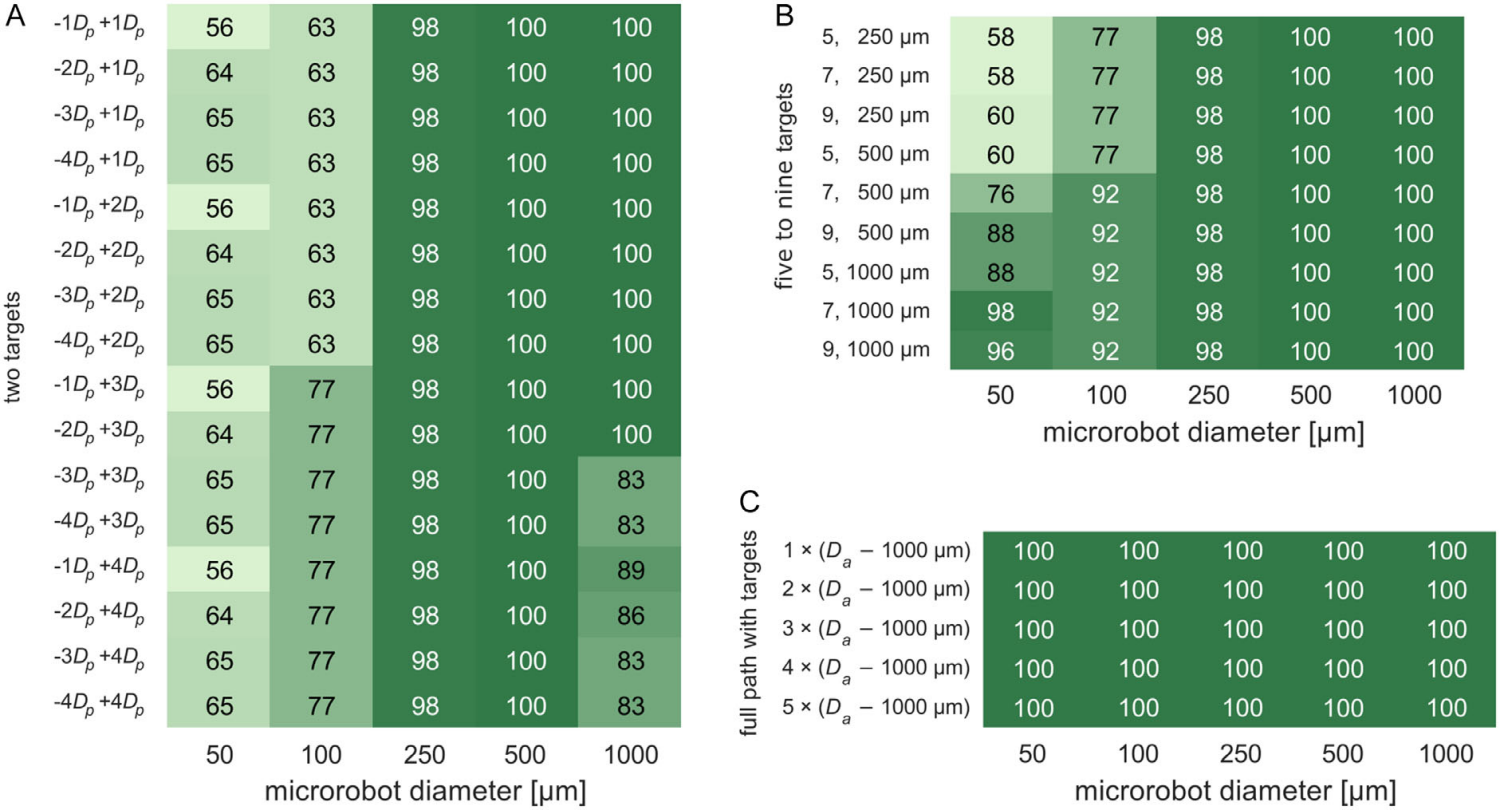
Navigation success [%] when steering microrobots of five different diameters (50–1000 μm) with different number and positioning of the targets in each of the three navigation strategies considered in this work (*n* = 96 for each table cell): A) Two‐target strategy, B) five‐to‐nine‐target strategy, and C) full path with target strategy. In this analysis, the magnetic gradient imposed on the microrobots is updated at every time step as they move through the neurovascular network. The navigation success was calculated as the ratio between the number of microrobots reaching the target vessels and the number of microrobots released at the inlets (i.e., at the different 16 entrance positions considered, Figure S2B, Supporting Information).

The navigation success [%] was calculated as the ratio between the number of microrobots reaching the last downstream target positioned in the desired vessels (where clots might be), and the total number of microrobots released at the different inlets of the neurovascular network. The tables in **Figure** **4** show the navigation success for the five microrobot diameters when considering the different numbers and positioning of the targets, in the three different navigation strategies studied in this work. In general, the two‐target strategy allows to successfully navigate the vast majority of the larger microrobots (*D*_*p*_ of 250, 500, and 1000 μm; Figure 4A) along the six routes considered in this work (Figure 1). This is particularly true for microrobots with diameters of 250 and 500 μm, for which the navigation was successful in 98 % and 100 % of the simulation scenarios, respectively, for all the tested positions of the upstream and downstream targets (i.e., between –4*D*_*p*_ to +4*D*_*p*_). Microrobots with a diameter of 1000 μm can also be successfully navigated for all the tested positions of the upstream target, provided that the downstream target is placed no more than two microrobot diameters (2*D*_*p*_) away from the point of flow splitting (i.e., target positions from –1*D*_*p*_ +1*D*_*p*_ to –4*D*_*p*_ +2*D*_*p*_, Figure 4A). Moreover, the downstream target can even be placed 3*D*_*P*_ away from the point of flow splitting so long as the upstream target is placed 1*D*_*p*_ to 2*D*_*p*_ away from the point of flow splitting (i.e., –1*D*_*p*_ +3*D*_*p*_ and –2*D*_*p*_ +3*D*_*p*_, Figure 4A). But even if targets are not placed in the aforementioned ideal positions, successful navigation can still be achieved in 83–89% of the remaining simulation scenarios (i.e., –3*D*_*p*_ +3*D*_*p*_ to –4*D*_*p*_ +4*D*_*p*_, Figure 4A), which is nevertheless an interesting result. Furthermore, that the position of the targets is more relevant in the navigation of the larger microrobots is not surprising because their larger size leads to more collisions with the artery walls, and thus to deviations from the ideal trajectory and loss of momentum due to the low flow velocities near the walls. Because of this, as a general rule, when navigating these large microrobots, it is important to position the downstream target up to two microrobot diameters away from the point of flow splitting, to minimize the likelihood of the microrobots colliding and getting trapped near the walls (Figure S3, Supporting Information).

The navigation of the smaller microrobots, that is, those with diameters of 50 and 100 μm, is more challenging than that of the larger microrobots. The navigation was successful in 56–65% of the simulation scenarios for the 50 μm microrobots, and in 63–77% of the scenarios for the 100 μm microrobots (Figure 4A, *D*_*p*_ of 50 and 100 μm). Moreover, the position of the targets has limited effect over the navigation success of these two small microrobots, with the navigation success being 8–9 percentage points higher with upstream targets farther away from the flow splitting for the 50 μm microrobots (i.e., −2*D*_*p*_, –3*D*_*p*_, and −4*D*_*p*_, Figure 4A), and 14 percentage points higher with downstream targets farther away from the flow splitting (i.e., +3*D*_*p*_ and +4*D*_*p*_, Figure 4A) for the 100 μm microrobots. Navigating these smaller microrobots is more challenging for two reasons: 1) smaller microrobots get closer to the artery walls than larger microrobots, reaching regions with much lower flow velocity due to the prevailing no‐slip condition at the walls, and 2) smaller microrobots are more strongly affected by the blood‐induced drag because the drag force decreases less than the magnetic force when the microrobot diameter decreases (the drag force scales with the microrobot surface area, whereas magnetic force scales with the microrobot volume; Equation (5)). For these reasons, smaller microrobots are more easily decelerated when reaching regions with low flow velocity, thus getting more easily trapped at the walls, particularly when the direction of the magnetic gradient points somewhat toward the wall (Figure S3 and S5, Supporting Information). As a result, the navigation success for the two smaller microrobot diameters (56–65% and 63–77% [50 and 100 μm], Figure 4A) was much lower than that for the largest three microrobot diameters (98%, 100%, and 83–100%, [250, 500, and 1000 μm], Figure 4A). Nevertheless, and despite the observed trapping of some of the smaller microrobots, the two‐target strategy is quite appealing given its simplicity and the ability to successfully navigate microrobots with diameters ≥250 μm along the six studied routes (Figure 1) in ≥98% of the simulation scenarios provided that, for the largest microrobots, the downstream target is placed within two microrobot diameters of the point of flow splitting (Figure 4A).

Having observed that using two targets per bifurcation does not warren a successful navigation for all the tested simulation scenarios, particularly for the smaller microrobots (i.e., *D*_*p*_ of 50 and 100 μm), we focused on the five‐to‐nine‐target strategy implying the use of five, seven, and nine targets per bifurcation, that is, two, three, and four upstream and downstream the point of flow splitting, separated by 250, 500, and 1000 μm. Given the higher number of targets per bifurcation, we expected more microrobots to be successfully navigated along the six studied routes. As expected, with the five‐to‐nine‐target strategy, higher navigation success was obtained for all tested microrobot diameters (Figure 4B versus Figure 4A), specifically in 98%, 100%, and 100% of the simulation cases, for the microrobot diameters of 250, 500, and 1000 μm, respectively (Figure 4B). Importantly, these high values of navigation success were obtained for all the different cases considered, which means that using five to nine targets per bifurcation, spaced between 250 and 1000 μm, offers very strong indications that the larger microrobots (250, 500, and 1000 μm) can be successfully navigated along the considered six routes. Higher navigation success was also observed for the smaller microrobots (50 and 100 μm). While the two‐target strategy led to successful navigation in 56–65% and 63–77% of the simulation scenarios for the 50 and 100 μm microrobots (Figure 4A), the five‐to‐nine‐target strategy led to successful navigation in 58–98% and 77–92% of the simulation scenarios (Figure 4B). More importantly, when using the five‐to‐nine‐target strategy with the 50 μm microrobots, navigation success was obtained in >96% of the simulation scenarios when positioning seven targets 1000 μm apart, and in the case of the 100 μm, navigation success >92% was obtained when positioning seven or more targets at least 500 μm apart (or five targets 1000 μm apart, Figure 4B). Overall, these results show that the higher number of targets and the larger intertarget distance of five‐to‐nine‐target strategy allow for a better steering of the smaller microrobots along the bifurcations, and much less trapping of the microrobots in regions of low flow velocity near the artery walls.

Finally, when using the strategy with the full path covered with targets, successful navigation was achieved in 100% of the simulation scenarios, for every microrobot diameter and intertarget distances considered (Figure 4C). As expected, having targets spread along the entire length of the routes allowed for a smooth correction of the microrobots trajectory as they move along the bifurcations, even if/when they deviated from course or collided with the artery walls. This was particularly relevant for the smaller 50–100 μm microrobots because it avoided their trapping near the walls, as observed in some scenarios when using the mentioned two targets and the five‐to‐nine‐target strategies (Figure S3, Supporting Information).

**Figure 5:**
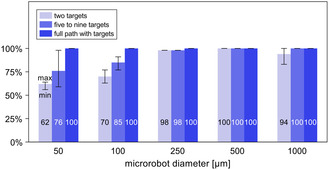
Average navigation success obtained for each navigation strategy considering all the different cases (with different numbers and positioning of the targets), when the magnetic gradient is updated at every time step. The values displayed inside each bar correspond to the average navigation success obtained for the considered microrobot diameter and navigation strategy (*n* = 1536, 864, and 480 in each bar for the two targets, five to nine targets, and full path with target strategy, respectively). The horizontal lines below and above each bar top represent the minimum and maximum navigation success, respectively, obtained for the considered microrobot diameter and navigation strategy.

The results above show that it is possible to successfully navigate magnetic microrobots with a wide range of diameters through a patient‐specific neurovascular network by using magnetic gradients updated at every new position of the microrobots. The tested three navigation strategies led to successful navigation for the vast majority of microrobot diameters considered in this work (**Figure** 4A–C and **5**), even if requiring, particularly for the smaller microrobot diameters, careful positioning of the intermediate targets in the vicinity of bifurcations (Figure 5).

### 3.2 Navigation with Spatially Constant Magnetic Gradients

The magnetic gradients described in the previous section were calculated based on the position and velocity of the microrobots as they progressed through the neurovascular network. This iterative calculation implied the update of the magnetic gradients at every time step, depending on the exact position and velocity of the microrobots. Replicating this almost “continuous” updating of the magnetic gradients in clinical environments is not practical because it would require magnetic manipulation at very high frequencies. More importantly, it would not be safe for the patient because imaging the neurovascular network at such high frequency (for spotting the microrobot positions) would expose the patient to considerable radiation (note that typical C‐arms usually operate at no more than 30 Hz).^[^ ^72^, ^73^ ^]^ Yet, it may be possible to avoid the need for imaging the patient and updating the magnetic gradients at high frequencies by relying instead on spatially constant magnetic gradients that may still successfully navigate the microrobots.

**Figure 6:**
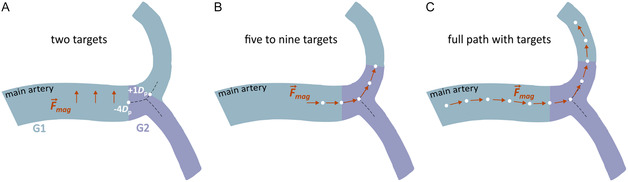
Regions considered for the spatially constant G1 and G2 regions in a general bifurcation. The G1 region (green/blue) corresponds to the portion of the bifurcation upstream of the last upstream target, whereas the G2 region (purple) corresponds to the portion of the bifurcation from the last upstream target to the last downstream target. In general, the region downstream of the last downstream target is the G1 region of the next bifurcation. For defining the G1 and G2 regions for the full path with target strategy, the artery connecting two consecutive bifurcations is assumed to be divided in half between the two bifurcations, with the first half assumed to belong to the first bifurcation, and the second assumed to belong to the second bifurcation. For each navigation strategy considered in this work, the number and position of the intermediate targets (white dots) and the different directions of the magnetic force (orange arrows) are represented: A) Two‐target strategy for which the magnetic force is perpendicular to the main artery. B,C) Five to nine targets and full path with target strategies, for which the magnetic force points from one target to the next.

In our previous work on the navigation of microrobots along ideal bifurcations,^[^ ^24^ ^]^ we observed that the magnitude of the magnetic gradients clustered around two distinctive values, one in the region upstream the point of flow splitting (G1, blue region, **Figure** **6**) and another in the region downstream the point of flow splitting (G2, purple region, Figure 6). Based on this observation, we hypothesized that the same would occur in the bifurcations of the considered neurovascular network. For that reason, having calculated the required magnetic gradient magnitudes for all the 14400 simulation scenarios, we averaged, for each microrobot diameter and manipulation strategy, the magnitudes obtained upstream and downstream the point of flow splitting in each bifurcation, to generate spatially constant G1 and G2 magnetic gradient magnitudes that can represent the typical magnetic gradients required in the G1 and G2 regions (Figure 6). If proven adequate, these spatially constant G1 and G2 gradients could be used to navigate the microrobots along each route without the need for updating the gradients at every new incremental position of the microrobots. As mentioned above, this is important for the development of manipulation technologies and for their clinical translation because 1) imposing spatially constant magnetic gradients that require updating only when a microrobot reaches a new target, rather than at every new incremental position of the microrobot, is less demanding on the manipulation technology, because the magnetic gradients are updated at lower frequencies, and 2) it is safer for the patients because spotting when a microrobot reaches a new target requires far less frequent imaging and exposure of the patients to imaging radiation, than one requiring the spotting of every new incremental position of the microrobot.

**Figure 7:**
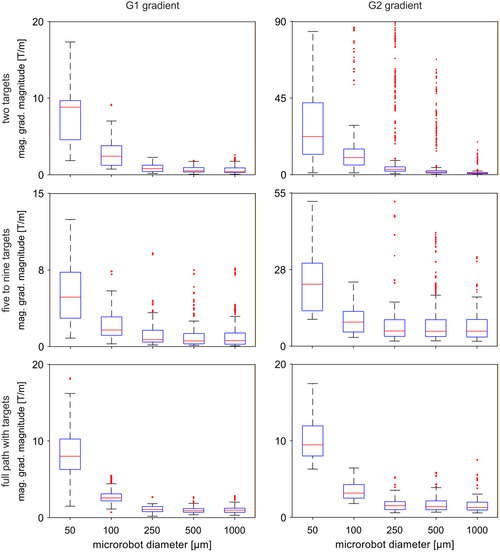
Boxplots for the G1 and G2 average gradient magnitudes obtained for each microrobot diameter using the three navigation strategies considered in this work (*n* = 2880 in each group). Each blue rectangle represents the interquartile range, containing 50% of the data; the bottom/top lines of the rectangle correspond to the lower/upper quartiles, where 25% of the data points lie below and above, respectively; the red line represents the median value; the top/bottom black lines represent the maximum/minimum values that fall outside 1.5× the value of the interquartile range, above/below the upper/lower quartiles; the red dots represent the outliers, which are higher/lower than the maximum/minimum.

To test the possibility of navigating the microrobots along the six cerebral routes using only spatially constant magnetic gradients, we re‐ran the simulation scenarios but now considering the G1 and G2 magnetic gradients in their corresponding regions (Figure 6). First, we obtained the averaged G1 and G2 magnetic gradients in their respective regions, for every bifurcation that was successfully navigated in the different simulation scenarios (i.e., the bifurcations where the microrobots were able to reach the last downstream target). This generated between 1 and 3 pairs of G1 and G2 magnetic gradients for each simulation scenario, depending on the number of bifurcations at each cerebral route (i.e., 2–3, Figure 1), and whether all bifurcations in each route were successfully navigated. The obtained spatially constant G1 and G2 magnetic gradient magnitudes were grouped according to the microrobot diameter for each of the three manipulation strategies and statistically analyzed using boxplots (**Figure** **7**). The boxplots allow to observe the variability and skewness of the data and to compare the spatially constant G1 and G2 gradient magnitudes for the different microrobot diameters. The blue rectangle in the boxplots (Figure 7) represents the interquartile range where 50% of the data points lie, with the top and bottom lines representing the lower and upper quartiles, the values at which 25% of the data sit above or below, respectively. The red line represents the median data value while the top and bottom black lines represent the maximum/minimum values that fall outside 1.5× the value of the interquartile range, above/below the upper/lower quartiles. The outliers, which are higher/lower than the maximum/minimum values, are represented by the red dots.

Figure 7 shows that, for the three navigation strategies considered in this work, the interquartile range and the median of the G1 and G2 gradient magnitude sharply decrease with increasing microrobot diameter, meaning that the microrobot diameter has a strong effect on the gradient magnitude. Additionally, the distinctive median value of the gradient magnitudes for each microrobot diameter (horizontal red lines in Figure 7) shows that the median value may be a good predictor of the overall G1 and G2 gradient magnitudes required for successful navigation along the neurovascular network. Finally, the small interquartile range displayed for microrobots with diameters ≥250 μm indicates low variability, meaning that the required G1 and G2 gradient magnitudes are similar across the different routes. This indicates that using median G1 and G2 gradient magnitudes may be adequate for successfully navigating the microrobots along all tested routes. Based on these insights, we calculated the median of the spatially constant G1 and G2 gradient magnitudes for each navigation strategy and used them in a data‐driven modeling approach to generate equations that can predict the median G1 and G2 magnitudes based on the microrobot diameter (Figure S4, Supporting Information). These second‐order linear equations, obtained using the least‐squares method^[^ ^74^ ^]^ for each navigation strategy, are useful because they can be easily implemented in algorithms automating the updating of the magnetic gradients based on the microrobot size and position in each bifurcation.

The averaging approach described for generating the G1 and G2 gradient magnitudes cannot be applied for processing the values of the gradient direction, because the latter very much depends on the route the microrobots must navigate, which may require opposite motions. For instance, different routes may require the microrobots to move in opposite directions at the same bifurcation (e.g., the two routes in Figure 1A, B, or C), and thus, using a median value of the gradient direction would not lead the microrobots along the desired trajectory. For this reason, in the testing of the spatially constant G1 and G2 gradient magnitudes, we set the G1 and G2 gradient direction depending on the chosen navigation strategy and on the position of the targets (Figure 6). Specifically, for the two‐target strategy, both G1 and G2 gradient directions were set to be perpendicular to the main artery (pointing toward the bifurcation branch containing the downstream target, Figure 6A), to maximize the magnetic force acting of the microrobot in that direction. For both the five to nine targets and the full path with targets strategies, the G1 and G2 gradient directions were set to point from one target to the next, leveraging the larger number of targets to better steer the microrobots along the bifurcation curvatures (Figure 6B–C).

Having generated equations predicting the median spatially constant G1 and G2 gradient magnitudes for each microrobot diameter (Figure S4, Supporting Information), and having set the direction of the gradient magnitudes for the three navigation strategies (Figure 6), we re‐ran all the simulations for testing the possibility of navigating the microrobots along the six cerebral routes (Figure 1) using only the spatially constant G1 and G2 gradient instead of the specific magnetic gradients updated at every time step (Section 3.1). We then proceeded with the evaluation of the navigation success by checking the number of microrobots reaching the last downstream target in the desired vessels (assumed to be near a clot) in comparison with the total number of microrobots entering the neurovascular network at the defined inlets. Unlike the initial analysis (Sections 2.5 and 3.1) where we considered the release of microrobots from 16 different representative positions at each inlet (Figure S2B, Supporting Information), here we strongly widened the number of positions from where microrobots could be released, to better account for what may happen in a clinical environment, where microrobots may occupy any radial and angular position inside the vessels when entering the neurovascular network. Specifically, in each of the three inlets (Figure 1), we assumed that microrobots were released from the centers of all mesh elements that were at least one microrobot radius away from the wall (in line with our strategy to improve the accuracy in the prediction of the microrobots trajectory, Section 2.2.4; Figure S2C, Supporting Information). This resulted in the release of microrobots from 279 to 1754 different positions at each inlet (depending on the diameters of the microrobots and the inlets, since the center of larger microrobots have less space available to move radially inside the arteries, and bigger inlets have more mesh elements from which the microrobots can be released; Table S1, Supporting Information). Based on this, we tested the navigation of the microrobots using the spatially constant G1 and G2 gradient magnitudes for a total of 916800 simulation scenarios (i.e., 30560 release positions × 30 navigation strategies, considering the 6 routes and 5 microrobot diameters, Table S1, Supporting Information).

**Figure 8:**
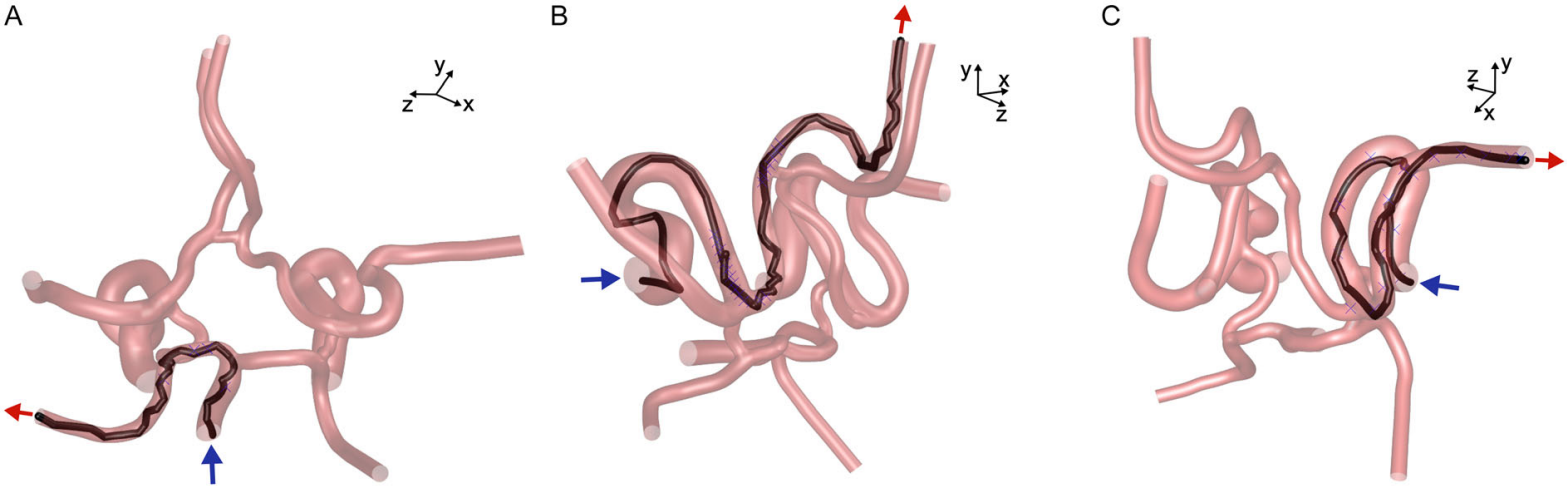
Examples of successful navigation of three microrobots (1000 μm) through three different routes, using each of the three navigation strategies based on spatially constant G1 and G2 magnetic gradients. In each route, the inlet and the outlet are represented by the blue and red arrows, respectively. The microrobots are represented by their spherical volume throughout their trajectory, and the intermediate targets are represented by the blue crosses. A) Navigation of a microrobot entering the BA and steered to the LPCA outlet using the two‐target strategy, with targets placed four microrobots diameter upstream and downstream the position of flow splitting in each bifurcation. B) Navigation of a microrobot entering the LICA and steered to the LACA using the five‐to‐nine‐target strategy with seven targets, 1000 μm apart in each bifurcation. C) Navigation of a microrobot entering the RICA and steered to the RMCA using the full path with target strategy with the targets separated by five times the radial distance available for the 1000 μm microrobot to move inside each artery without touching the walls (i.e., 5 × (*D*_*a*_−1000 μm) in Table 2).

Examples of successful navigation through three of the six routes (i.e., BA to LPCA, LICA to LACA, and RICA to RACA) using the spatially constant G1 and G2 magnetic gradients are shown in **Figure** **8**, for a microrobot with a diameter of 1000 μm represented along each trajectory by its volume.

**Figure 9:**
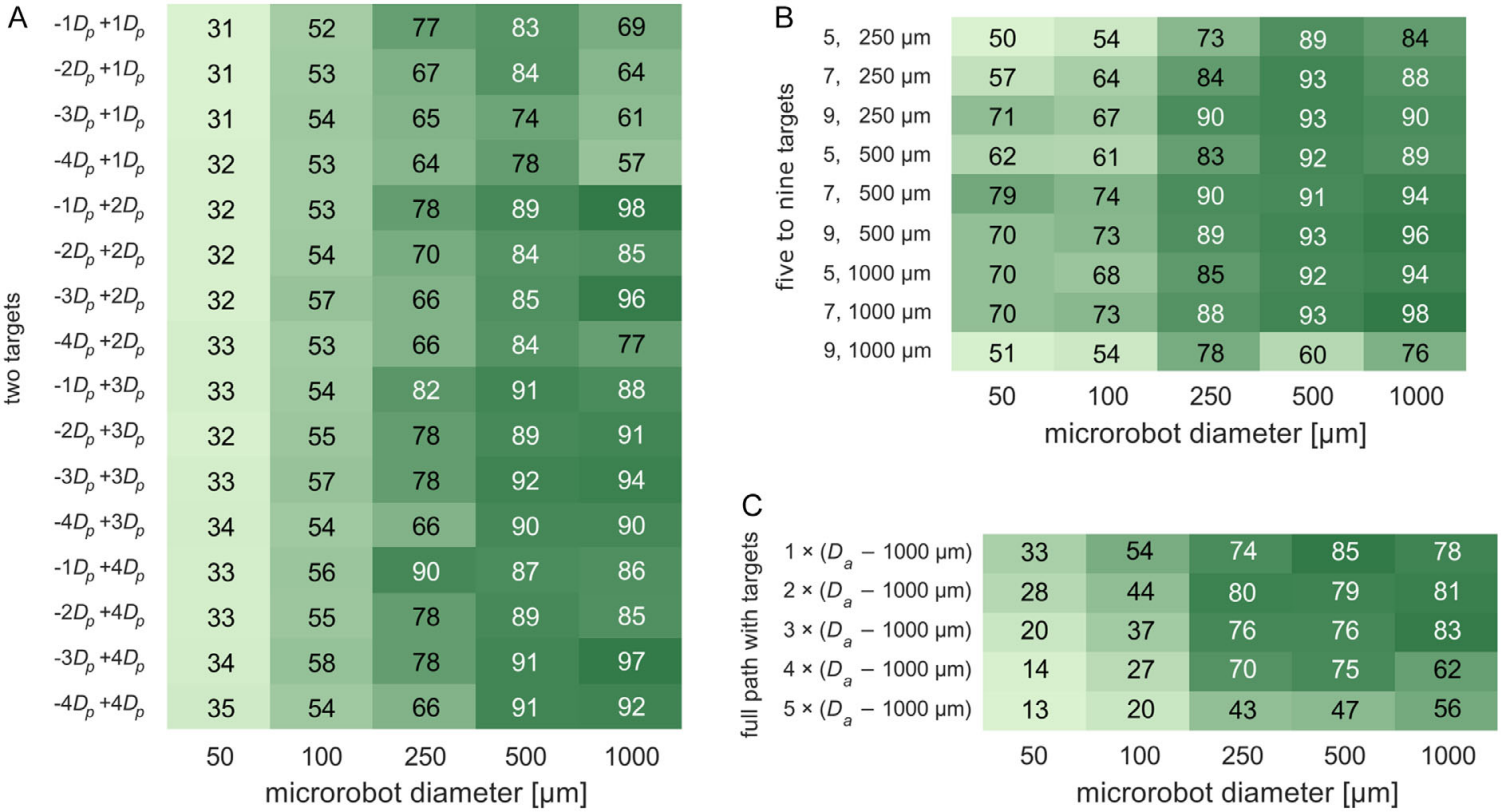
Navigation success [%] when steering microrobots of five different diameters (50–1000 μm) with the different number and positioning of the targets in each of the three navigation strategies considered in this work: A) Two‐target strategy, B) five‐to‐nine‐target strategy, and C) full path with target strategy. In this analysis, the magnetic gradient imposed on the microrobots is updated only when microrobots reach a new target, and the gradient magnitude is equal to the median value of the spatially constant G1 and G2 magnetic gradient magnitudes, obtained in the G1 and G2 regions (Figure 6). The navigation success was calculated as the ratio between the number of microrobots reaching the target vessels and the number of microrobots released at the inlets (i.e., at the 279–1754 different entrance positions considered, which depend on the inlet and microrobot diameters, Table S1, Supporting Information). In each table cell, *n* = 9024, 8236, 5196, 5236, and 2868 for the microrobot sizes of 50, 100, 250, 500, and 1000 μm, respectively.

**Figure** **9** shows the navigation success obtained for each navigation strategy and microrobot diameter, when using the spatially constant G1 and G2 magnetic gradients. In general, the navigation success obtained using the spatially constant G1 and G2 magnetic gradients is somewhat lower than that obtained when updating the magnetic gradients at every time step. This is not surprising given the much higher number of release positions considered (30560 now versus 16 before), and the use of spatially constant G1 and G2 values that are updated only when the microrobots moves from one region to the next (from G1 to G2, Figure 6). However, despite the more demanding testing conditions, the obtained navigation success is very promising, with the vast majority of microrobots with diameters ≥250 μm reaching the desired target (Figure 9). The two‐target strategy allowed to navigate microrobots with diameters of 250, 500, and 1000 μm in 64–90%, 74–92%, and 57–98% of the simulation scenarios, respectively. Furthermore, microrobots with diameter of 250 μm were successfully navigated in 90% of the scenarios when placing the targets in the –1*D*_*p*_ +4*D*_*p*_ positions. More importantly (given their potential for drug‐delivery), microrobots with diameters of 500 μm and 1000 μm were successfully navigated in 87–92% and 85–97% of the scenarios, respectively, when the downstream targets were placed three or four microrobot diameters away from the position of flow splitting (Figure 9A). As observed before, the navigation of the smaller microrobots (50 and 100 μm) was more challenging, with successful navigation being achieved in only 31–35% and 52–58% of the scenarios, and the specific target positions having limited influence. This was not surprising. Magnetic gradients perpendicular to the main arteries steer the microrobots closer to the walls, and that together with the fact that small microrobots are more easily decelerated in the regions with low flow velocity near the walls, likely contributed to the trapping of many microrobots near the walls (Figure S5, Supporting Information).

Using more targets upstream and downstream, the point of flow splitting generally increased the navigation success across all microrobot diameters, as shown by the results for the five‐to‐nine‐target strategy (Figure 9B). Microrobots with diameters of 250, 500, and 1000 μm were successfully navigated in 73–90%, 60–93%, and 76–98% of the simulation scenarios, respectively (Figure 9B). Moreover, navigation success >85%, >90%, and >95% proved to be achievable, for the diameters of 250, 500, and 1000 μm, respectively, with many of the tested number of targets and intertarget distances (e.g., 7 targets 500 or 1000 μm apart, or 9 targets 250 or 500 μm apart, Figure 9B). The navigation of the microrobots with diameters of 50 and 100 μm was also far more successful when using the five‐to‐nine‐target strategy, leading to navigation success in 50–79% and 54–74% of the scenarios (Figure 9B), which compare favorably with 31–35% and 52–58% obtained with the two‐target strategy (Figure 9A). To this improved navigation of the microrobots contributed not only the higher number of targets placed in the vicinity of the bifurcations (Figure 2B), but also the imposition of magnetic gradients pointing from one target to the next (Figure 6B), which decreased the likelihood of microrobots reaching near‐wall regions that trapped many microrobots when using the two‐target strategy.

**Figure 10:**
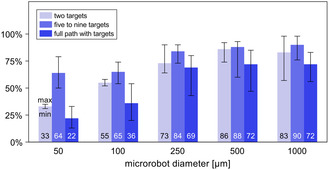
Average navigation success obtained for each navigation strategy considering all the different cases (i.e., different number and positioning of the targets), when imposing spatially constant G1 and G2 magnetic gradients. The values displayed inside each bar correspond to the average navigation success obtained for the considered microrobot diameter and navigation strategy. The horizontal lines below and above each bar top represent the minimum and maximum navigation success, respectively, obtained for the considered microrobot diameter and navigation strategy. For each bar, the value of *n* depends on the number of cases (16, 9, and 5, Table 2) considered for each navigation strategy, and the number of positions from which microrobots were released at each inlet (Table S1, Supporting Information) based on the microrobot diameter and artery type: for the two‐target strategy *n *= 144384, 131776, 83136, 83776, and 45888 for the microrobot sizes of 50, 100, 250, 500, and 1000 μm, respectively; for the five‐ to nine‐target strategy *n *= 81216, 74124, 46764, 47124, and 25812 for the microrobot sizes of 50, 100, 250, 500, and 1000 μm, respectively; and for the full path with target strategy *n *= 45120, 41180, 25980, 26180, and 14340 for the microrobot sizes of 50, 100, 250, 500, and 1000 μm, respectively.

Finally, the strategy with the full path covered with targets led to a lower navigation success compared to that obtained with the previous strategies (Figure 9C versus Figure 9A–B, and **Figure** **10**), and far less than that obtained when updating the gradients at every new position of the microrobots (Figure 9C versus Figure 4C). Across the five microrobot diameters, the navigation success obtained with the full path with targets is 8–46 percentage points lower than that obtained with the five‐to‐nine‐target strategy, highlighting the worse performance of the former. Having microrobots manipulated across the entire route rather than just at the bifurcations, with median magnetic gradients (from the equations in Figure S4, Supporting Information) that likely did not fully match what would be needed for them to follow the defined path (i.e., the gradients computed by Equation (8)), ultimately allowed a large number of microrobots to veer off course (Figure S5, Supporting Information). This had a larger influence over the navigation success of the smaller microrobots (*D*_*p*_ = 50 μm), and the manipulations relying on less targets placed farther apart (Figure 9C). As argued before, smaller microrobots are more easily trapped in regions of low flow velocity near the walls, and that occurs more often when the manipulation along curved vessels is done with targets placed farther apart. In the latter case, the direction connecting one target to the next, that is, the direction along which the magnetic gradient is imposed, may cross the artery walls, a situation where the gradient will ultimately pull the microrobots toward the walls, increasing the likelihood of trapping.

The results described previously show that it is possible to successfully navigate microrobots through the neurovascular network using spatially constant magnetic gradients, and that magnetic navigation is advantageous when compared with the results for no magnetic control (maximum 50% navigation success, Table S3, Supporting Information). Furthermore, they show that both the two‐target and the five‐to‐nine‐target strategies can be used to successfully navigate microrobots toward specific sites within the neurovascular network, thus offering health professionals different alternatives for locally delivering therapeutics directly to the target vessels. For instance, health professionals wishing to deliver drugs (e.g., thrombolytics in the case of ischemic stroke) in occluding a low‐complexity portion of a patient's neurovascular network may prefer the simpler two‐target strategy for navigating the microrobots. Alternatively, when wishing to deliver drugs in more complex portions of the neurovascular network, health professionals may prefer the five‐to‐nine‐target strategy, despite the more frequent imaging, for using the extra targets to better manage the more complex vasculature. The advantages and disadvantages of each navigation strategy must be evaluated to select the approach that ensures high navigation success and effective drug delivery while minimizing the required imaging frequency (and thus the patient and health professionals’ exposure to X‐rays radiation).

Furthermore, the navigation success associated with the tested navigation strategies and the equations predicting the required magnetic gradients (Equations S1–S6 and Figure S4, Supporting Information) provide relevant information when wishing to design microrobots and/or magnetic manipulation systems, for neurovascular navigation. This data establishes a quantitative relation between the properties of the microrobots (e.g., size, magnetization, and magnetic volumes) and the required magnetic gradients for successful navigation. Information on this relation is important for choosing the ideal properties of the microrobots and/or the magnetic manipulation systems being considered in different scenarios. And this information can be used in both directions, that is, for designing magnetic systems based on the properties of the microrobots, or for optimizing the latter based on the capacity of the magnetic systems we may have available.

Finally, the results obtained in this work show that the navigation success is higher for microrobots larger than 250 μm. This is convenient for two reasons. On the one hand, larger microrobots are potentially more interesting for drug delivery applications given their ability to carry higher loads of therapeutics. On the other hand, larger microrobots are easier to manipulate. The magnetic gradients required for navigating larger microrobots are orders of magnitude lower than those required for navigating smaller microrobots (e.g., G1 and G2 gradient magnitudes for navigating a 1000 μm microrobot are 8–22× and 4–26× lower, respectively, than those required to navigate a 50 μm microrobot; Figure 7 and S4, Supporting Information). Furthermore, lower gradient magnitudes can be imposed by smaller electromagnetic systems, that use lower currents and can be cooled by smaller cooling systems. This simplifies the development and reduces the potential cost of magnetic manipulation technologies compatible with clinical settings, thus helping to reduce barriers for adoption of these technologies by healthcare providers.

## 4 Conclusions

We numerically studied the navigation of magnetic microrobots in a patient‐specific neurovascular network, to identify how to optimize the navigation of microrobots toward various target vessels often obstructed in ischemic stroke patients. We investigated various navigation strategies, with different numbers and positioning of intermediate targets, for navigating microrobots with diameters of 50–1000 μm along various cerebral routes leading to the defined target vessels. We showed that it is possible to successfully navigate microrobots using spatially constant magnetic gradients and developed equations for predicting the required magnetic gradient magnitudes as a function of the microrobots diameter. We studied the potential of magnetic navigation systems for enabling the targeted delivery of therapeutics and described various navigation strategies that can be explored by health professionals in clinical settings.

## Conflict of Interest

The authors declare no conflict of interest.

## Supporting information

Supplementary Material

## Data Availability

The data that support the findings of this study are available from the corresponding author upon reasonable request.
