# Supplementary material for: Strategies for Navigating Magnetic Microrobots in Neurovascular Networks: A Numerical Analysis
Source: Small Sci. 2025 Jul 25;5(10):2500180. doi: 10.1002/smsc.202500180 (PMC12499408; doi:10.1002/smsc.202500180)
Supplement: Supplementary file 1 — Supplementary Material [file SMSC-5-2500180-s001.pdf]

Strategies for navigating magnetic microrobots in neurovascular networks: a numerical analysis

Pedro G Alves,[1,2] Maria Pinto,[3] Rosa Moreira,[3] Derick Sivakumaran,[4] Fabian Landers,[5] Maria Guix,[7] Bradley J Nelson,[5] Andreas D Flouris,[6] Salvador Pané,[5,*] Josep Puigmartí-Luis,[7,8,*] Tiago S Mayor[1,2,*]

[1] Transport Phenomena Research Centre (CEFT), Engineering Faculty, Porto University, Portugal
[2] Associate Laboratory in Chemical Engineering (ALICE), Engineering Faculty, Porto University, Portugal
[3] Experian, Portugal
[4] Magnebotix, Zurich, Switzerland
[5] Multi-Scale Robotics Lab, ETH Zurich, Zurich, Switzerland
[6] FAME Laboratory, Department of Exercise Science, University of Thessaly, Greece
[7] Depart. de Ciència dels Materials i Química Física and Institut de Química Teòrica i Computacional, University of Barcelona, Barcelona, Spain
[8] Institució Catalana de Recerca i Estudis Avançats (ICREA), Barcelona, Spain

* Corresponding authors: vidalp@ethz.ch; josep.puigmarti@ub.edu; tiago.sottomayor@fe.up.pt

Supplementary Information

Steady versus pulsatile blood flow

Across the literature, various works have concluded that steady state results can accurately approximate many hemodynamic parameters, especially when considering the time-average of those parameters. Flow characteristics such as the helicity, vorticity or the flow profile, [1–5] and parameters such as the blood velocity, [6] blood flow rate [7–9] and wall shear stress [6,10] have been analyzed by several studies to compare the difference between steady and pulsatile results. The similarity in steady and pulsatile flow patterns was observed by Malcom and Roach[5] and by Fukushima *et al*.[1] for bifurcations. Identical flow features for steady and pulsatile flows in cerebral aneurisms were observed by Mikhal *et al*.[3] and by Karmonik and co-workers,[2] who reported similar distributions of pressure, helicity, vorticity, and velocity, but with different scaling factors. Chen[7] and Hillen *et al*.[8] obtained similar flow rates in the cerebral circulation with and without pulsatility. Fahy *et al*.[9] studied the *in vitro* blood flow distribution in the Circle of Willis and showed a good prediction using steady flow when compared to pulsatile flow. For the capture of particles in pulsatile flow, the results of Berselli *et al*.[11] show that the capture region remains the relatively the same when the particles are released over the pulsatile flow period at different time instants. Also, the capture efficiency calculated at those different time instants showed low variability. Both results show that using time-averaged steady flow conditions allows to obtain good approximation to results obtained

using pulsatile flows. In an *in vitro study,* Bushi and co-workers[12] found that the distribution of 1.6 mm particles into a bifurcation with daughter branches of different sizes did not differ when using steady and pulsatile flow.

This survey of various works shows that we can use steady flow conditions to simulate the blood flow distribution in the cerebral network and obtain results like those of pulsatile flows, leveraging the lower computational cost. Nevertheless, despite the mentioned insights from the cited literature, to show that the variation of the blood velocity in pulsatile conditions does not significantly affect the navigation success obtained when using the spatially-constant magnetic gradients, we have simulated again the 916800 simulations scenarios described in section 3.2 for two different blood velocities, i.e. those corresponding to the maximum and minimum blood flow rates observed for a typical pulse wave in the internal carotid and basilar arteries.[13,14] We first prepared pulse waves whose averages matched the flow rates considered in our simulations (Figure S6). From these pulse waves, we extracted the maximum and minimum blood flow rates to impose in our simulations as boundary conditions at the internal carotid and basilar arteries. Afterwards, we ran the mentioned simulations for the two blood flow rates to calculate the corresponding navigation successes (Figures S7 and S8) and used this data to obtain an estimate of the average navigation success over an entire cardiac cycle, by averaging the corresponding navigation successes. Finally, we compared the average navigation success obtained with the maximum and minimum blood flow rates of the cardiac cycle (i.e. average of the values from Figures S7 and S8), relative to the navigation success obtained with the average flow rate of a cardiac cycle (i.e. values in Figure 9, based on flow rates in Table 3), by subtracting the corresponding navigation success values (Figure S9). This comparison showed that the difference in the navigation success obtained in these two ways is not significant, being for most cases smaller than 15 percentage points (Figure S9). This confirms that the navigation success obtained in steady simulations based on the average flow rate of a cardiac cycle (i.e. data in Figure 9) is a good representation of the navigation success that would be obtained by averaging the navigation success corresponding to the different blood flow rates of the cardiac cycle.

Generalized form of the predictive equations

In this work, the calculated magnetic gradients were obtained for microrobots with a magnetic volume equal to the microrobot volume ($V_M = V_p$) and with a magnetization equal to the saturation magnetization ($M = M_S = 5\times10^5$ A·m^{-1}). These two parameters were chosen so that the calculated magnetic gradients would correspond to the minimum required to induce a given magnetic force (Equation 2). Nonetheless, for scenarios where the magnetic volume is less than the microrobot volume and/or the microrobot magnetization is less than the saturation value, the required magnetic gradients will be larger than the ones calculated by our predictive equations, since both parameters affect the predicted gradients in a linear inverse relationship. For this reason, the equations developed in this work for predicting the required magnetic gradients can be generalized by incorporating these inverse relationships:

Two Targets strategy

$$G1 = \left(0.44 - 16.74 d_p^{\;-1} + 21764.20 d_p^{\;-2}\right) \cdot \frac{V_p}{V_M} \cdot \frac{M_S}{M} \qquad \text{(S1)}$$

$$G2 = \left(-0.26 + 886.11{d_p}^{-1} + 12371.94{d_p}^{-2}\right) \cdot \frac{V_p}{V_M} \cdot \frac{M_S}{M} \tag{S2}$$

Five to Nine Targets strategy

$$G1 = \left(0.54 - 3.18{d_p}^{-1} + 10914.73{d_p}^{-2}\right) \cdot \frac{V_p}{V_M} \cdot \frac{M_S}{M} \tag{S3}$$

$$G2 = \left(5.53 - 217.51{d_p}^{-1} + 52646.86{d_p}^{-2}\right) \cdot \frac{V_p}{V_M} \cdot \frac{M_S}{M} \tag{S4}$$

Full Path with Targets strategy

$$G1 = \left(0.93 - 31.90{d_p}^{-1} + 19310.78{d_p}^{-2}\right) \cdot \frac{V_p}{V_M} \cdot \frac{M_S}{M} \tag{S5}$$

$$G2 = \left(1.34 - 40.82{d_p}^{-1} + 22360.20{d_p}^{-2}\right) \cdot \frac{V_p}{V_M} \cdot \frac{M_S}{M} \tag{S6}$$

Incorporation of the effect of interpatient differences in the numerical analyses

Blood flow rates in neurovascular regions vary across patients, for instance due to (1) interpatient differences in total cerebral blood flow and its distribution among cerebral arteries, and (2) anatomical variations in the geometry of the neurovascular network.

We incorporated the effect of interpatient differences in total cerebral blood flow rate and its distribution among cerebral arteries (point (1) above), by considering in our simulations average blood flow rates and average flow rate ratios (which dictate how the total cerebral blood flow rate is distributed to the various model inlets and outlets) based on the data from *in vivo* studies[15–18] characterizing the blood flow characteristics in the major cerebral arteries of 232 patients (Table 3; refs 13–16). This allowed to induce flow conditions in the neurovascular network that represent the average cerebral flow rates observed across a wide range of patients.

For mimicking the effect of anatomical variations in the neurovascular network geometry (point (2) above), we tested the navigation of the microrobots using magnetic gradients that were not specifically optimized for navigating along all the bifurcations contained in the considered neurovascular network. We did this because of the following reason. Although the results of this study show that spatially-constant magnetic gradients can be used to successfully navigate 50 – 1000 µm microrobots through the patient-specific neurovascular network considered here, one may wonder if this conclusion would hold for a different neurovascular network with distinct geometries or arterial arrangements. We posit that it would because applying magnetic gradients optimized for one vascular network to another, is analogous to using a bifurcation-specific gradient for navigating across different bifurcations. Both scenarios involve a mismatch between the imposed gradient and the theoretically optimal gradient for the target structure – whether an entire network or individual bifurcations. Importantly, because gradient mismatch did not preclude successful navigation through the

diverse bifurcations within the tested network, we believe that gradients optimized for our original network will remain effective in alternative networks, as the underlying physical principles governing magnetic navigation are consistent across vascular structures.

Supplementary Tables

Table S1 – Number of positions from which microrobots were released at each inlet, depending on the microrobot diameter and artery type (for the analysis of section 3.2). The microrobots were released from the center of every inlet face element that is one radius away from the artery wall, meaning that larger microrobots had less available space to be released from, but bigger arteries had more mesh elements from which microrobots could be released. A total of 30560 different release positions were considered (2 routes per inlet × number of release positions in each inlet).

		**Artery inlets**		
		**BA**	**LICA**	**RICA**
**Microrobot diameter [μm]**	**50**	1034	1754	1724
	**100**	1052	1674	1392
	**250**	636	1239	723
	**500**	563	1186	869
	**1000**	279	690	465

Table S2 – Time required for the microrobots to move along the neurovascular network [ms], for two microrobot diameters and three coefficients of restitution (COR), showing that smaller COR values delay the microrobots progression along the network.

		**Coefficient of Restitution (COR)**		
		**0.50**	**0.75**	**1.00**
**Microrobot diameter [μm]**	**50**	490	207	124
	**1000**	329	94	57

Table S3 – Navigation success [%] obtained for the microrobots of five diameters (50 – 1000 μm) when no magnetic force is applied, with the blood flow carrying the microrobots along the neurovascular network. The navigation success was obtained for a total of *n* = 30560 independent simulation cases.

	**Microrobot diameter [μm]**				
	**50**	**100**	**250**	**500**	**1000**
**Navigation success [%]**	38	34	40	42	50

Supplementary Figures

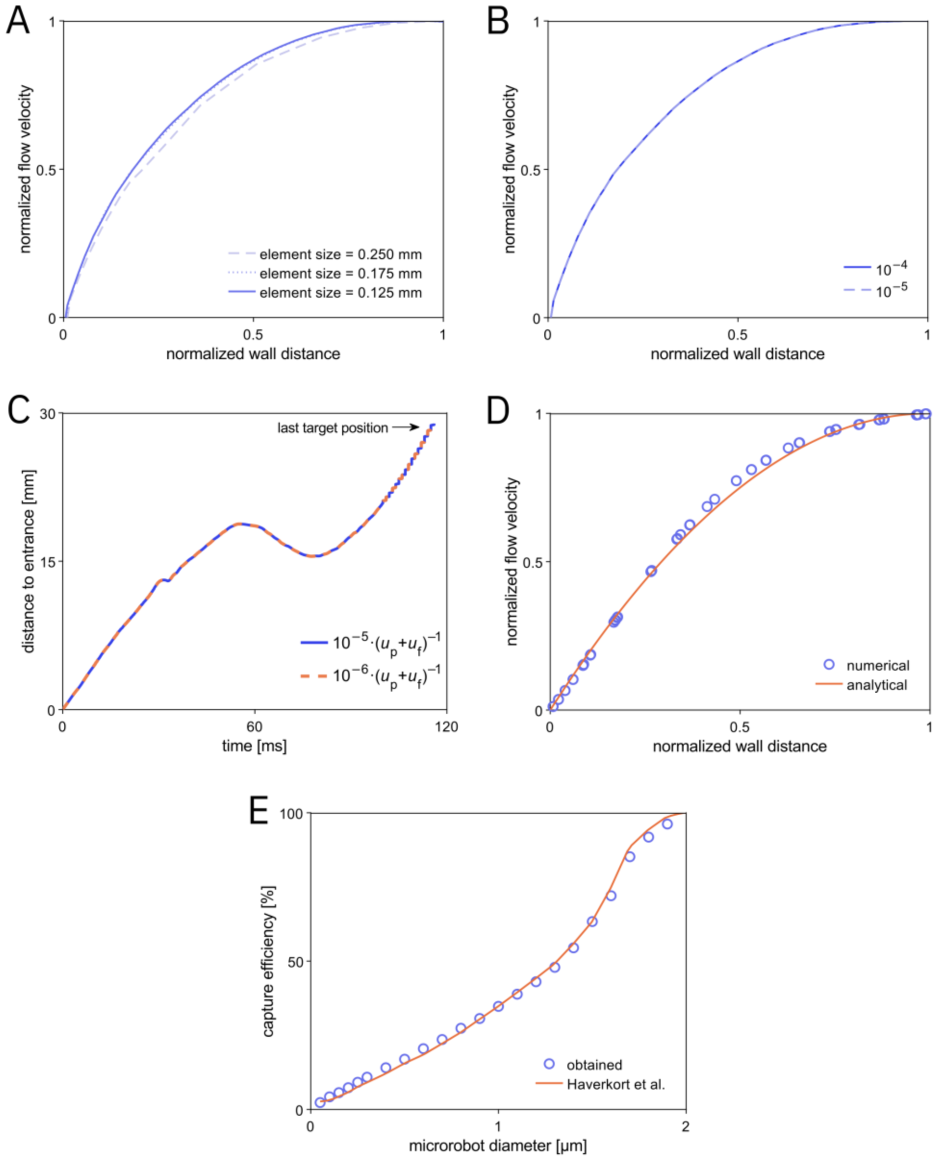

Figure S1 – Mesh independence tests, flow convergence criteria comparison, microrobot time step comparison and results obtained for the validation of the blood flow and magnetic navigation. A – Mesh independence tests considering three element sizes, for which a maximum element size of 0.175 mm produces mesh independent results. B – Normalized blood flows obtained using a convergence criterion of 10^{-4} and 10^{-5} for the continuity and velocity, showing that 10^{-4} is adequate, and that stricter criterion produces similar results. C – Comparison of microrobot trajectories (from entrance position to last target), obtained using time steps of $10^{-5}\cdot(u_p+u_f)^{-1}$ and $10^{-6}\cdot(u_p+u_f)^{-1}$, showing that using the former value is adequate since smaller time steps produce similar microrobot trajectories. E – Capture efficiency predicted by the present numerical approach and that reported by Haverkort *et al*.[19], for microrobots of different sizes.

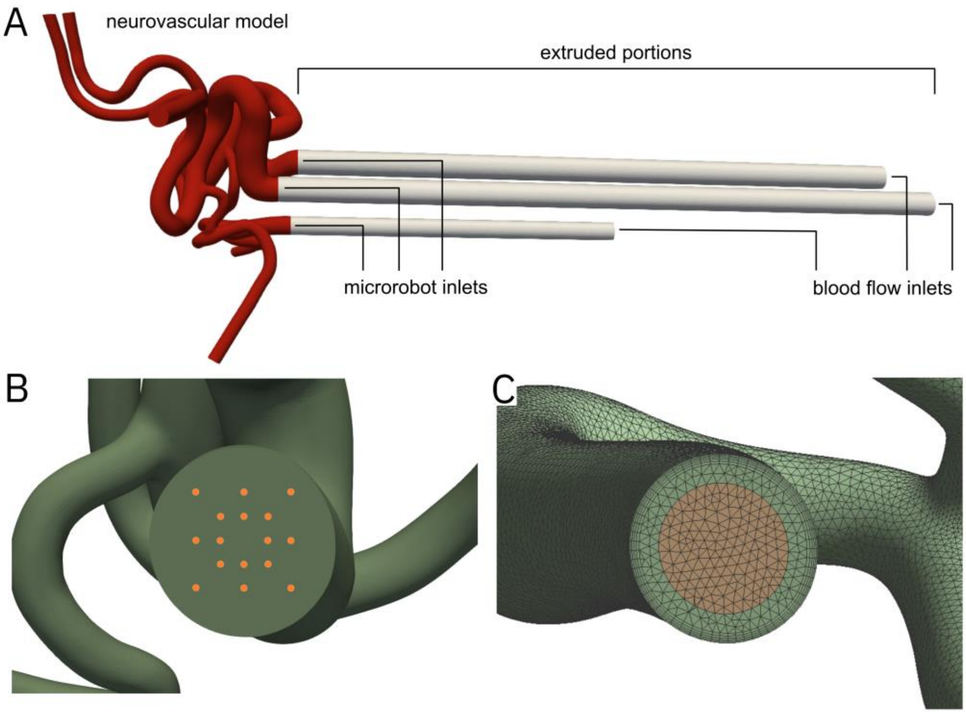

Figure S2 – Example of the neurovascular model and mesh elements used in this work. A – Neurovascular model (red) with the extruded portions (grey) that ensure that the blood flow imposed at the inlets is fully developed when it reaches the microrobot inlets. B – Sixteen representative entrance positions for the microrobots, placed radially at one-third and two-thirds between the inlet center and one microrobot radius away from the wall, and placed angularly at eight 45° intervals. C – Example of the 3D mesh used in the work, focusing on the face elements at the inlet that are one radius away from the wall (orange), and from where the microrobots are released (in the second set of 916800 simulations where the imposed magnetic gradient was spatially-constant in each of the G1 and G2 regions of the bifurcations, Figure 6, section 3.2).

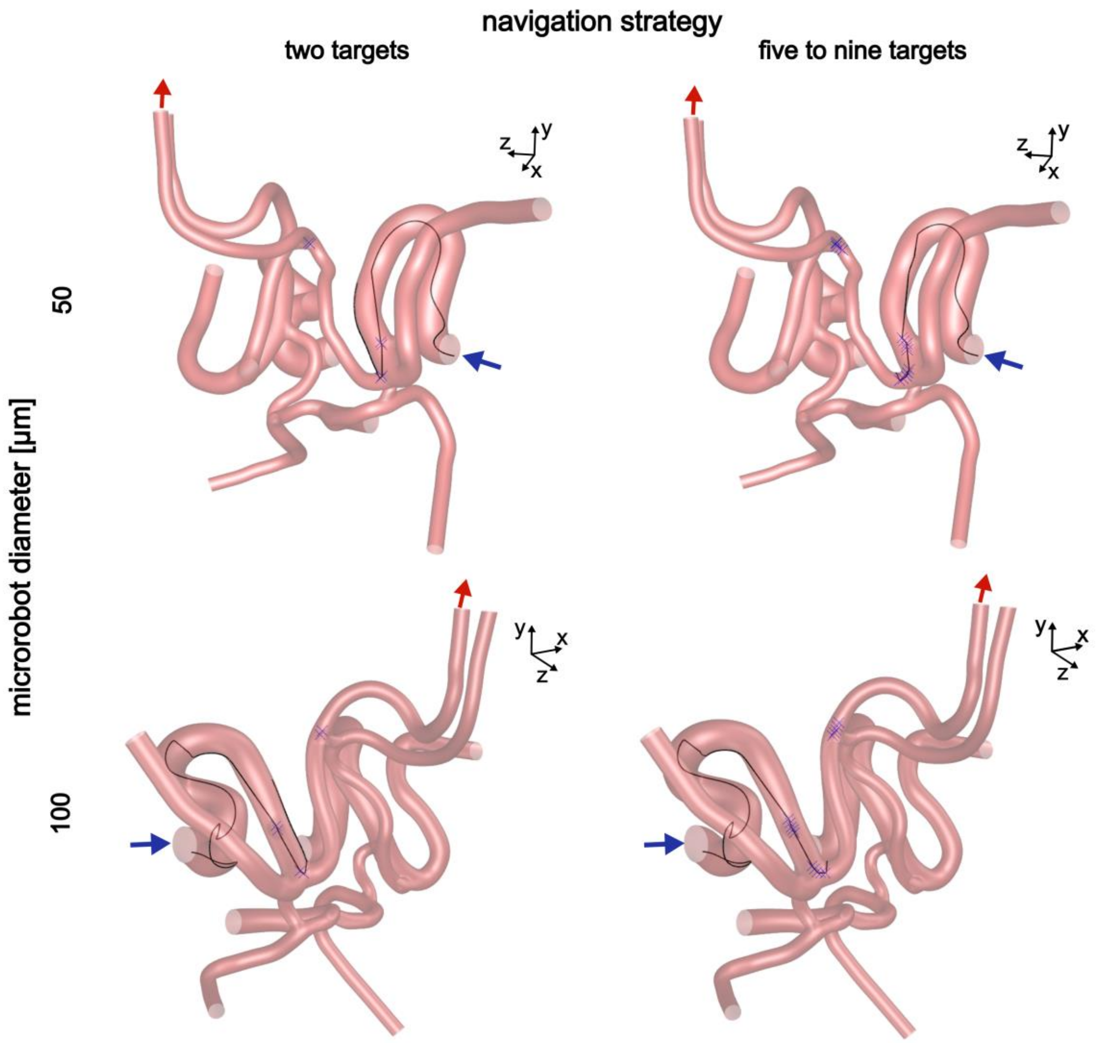

Figure S3 – Example of microrobots of diameter 50 and 100 µm that did not reach the target vessel due to becoming trapped at the artery walls. The 50 µm microrobot was navigated from the RICA to RACA using the two targets strategy, with targets placed four microrobot diameters upstream and downstream the position of flow splitting in each bifurcation; and using the five to nine targets strategy with five targets, 500 µm apart in each bifurcation. The 100 µm microrobot was navigated from the LICA to the LACA using the two targets strategy, with targets placed three microrobot diameters upstream and downstream the position of flow splitting in each bifurcation; and using the five to nine targets strategy with five targets, 500 µm apart in each bifurcation. The inlet and outlet associated with each route are represented by the blue and red arrows, respectively. The microrobots are represented by their spherical volume throughout their trajectory and the intermediate targets are represented by the blue crosses. The magnetic gradient used to navigate the microrobots is updated at every new incremental position.

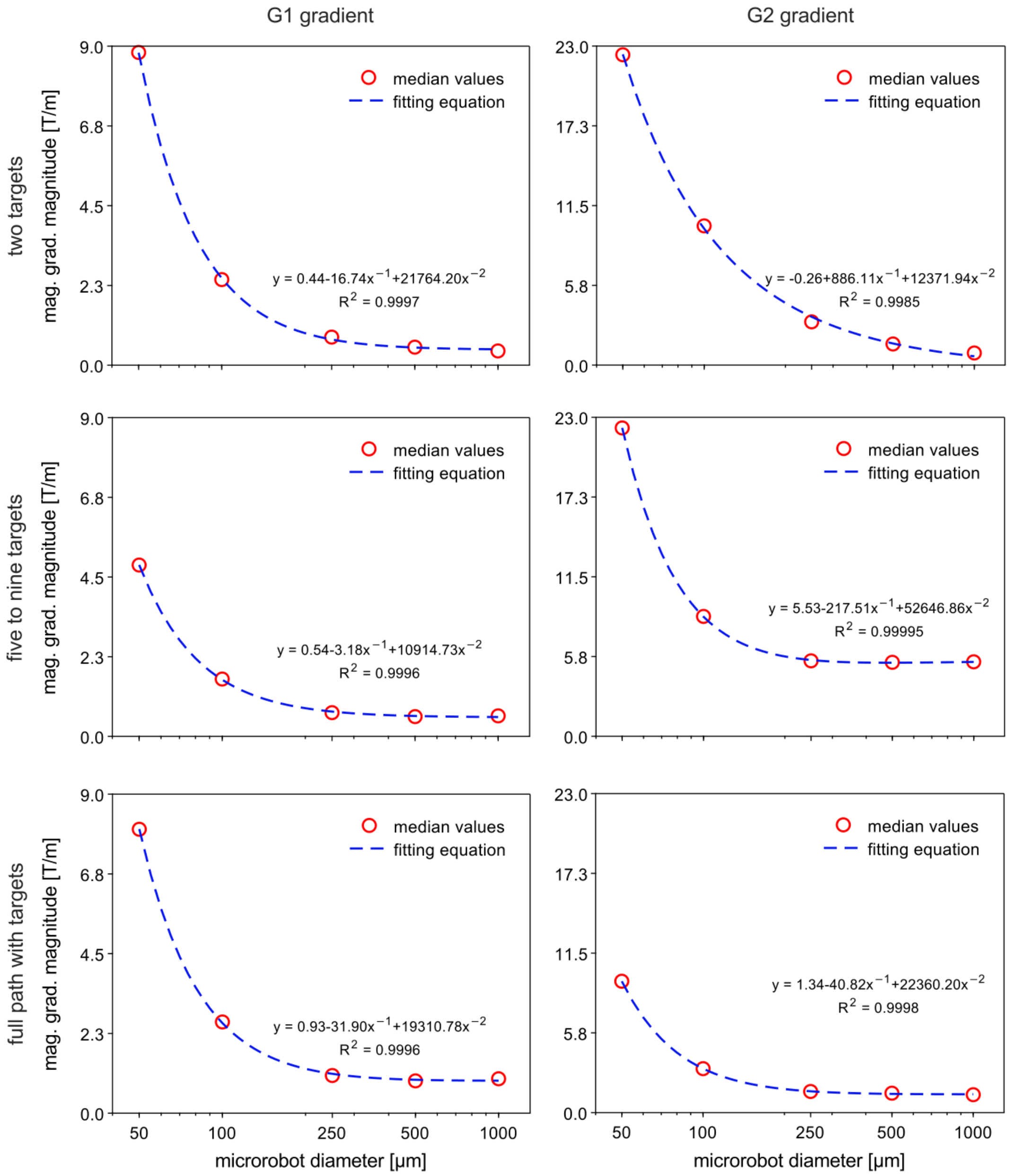

Figure S4 – Fitting equations obtained with the least-squares method (n = 5),[20] that can predict the median G1 and G2 gradient magnitudes based on the microrobot diameter, for each navigation strategy considered in this work.

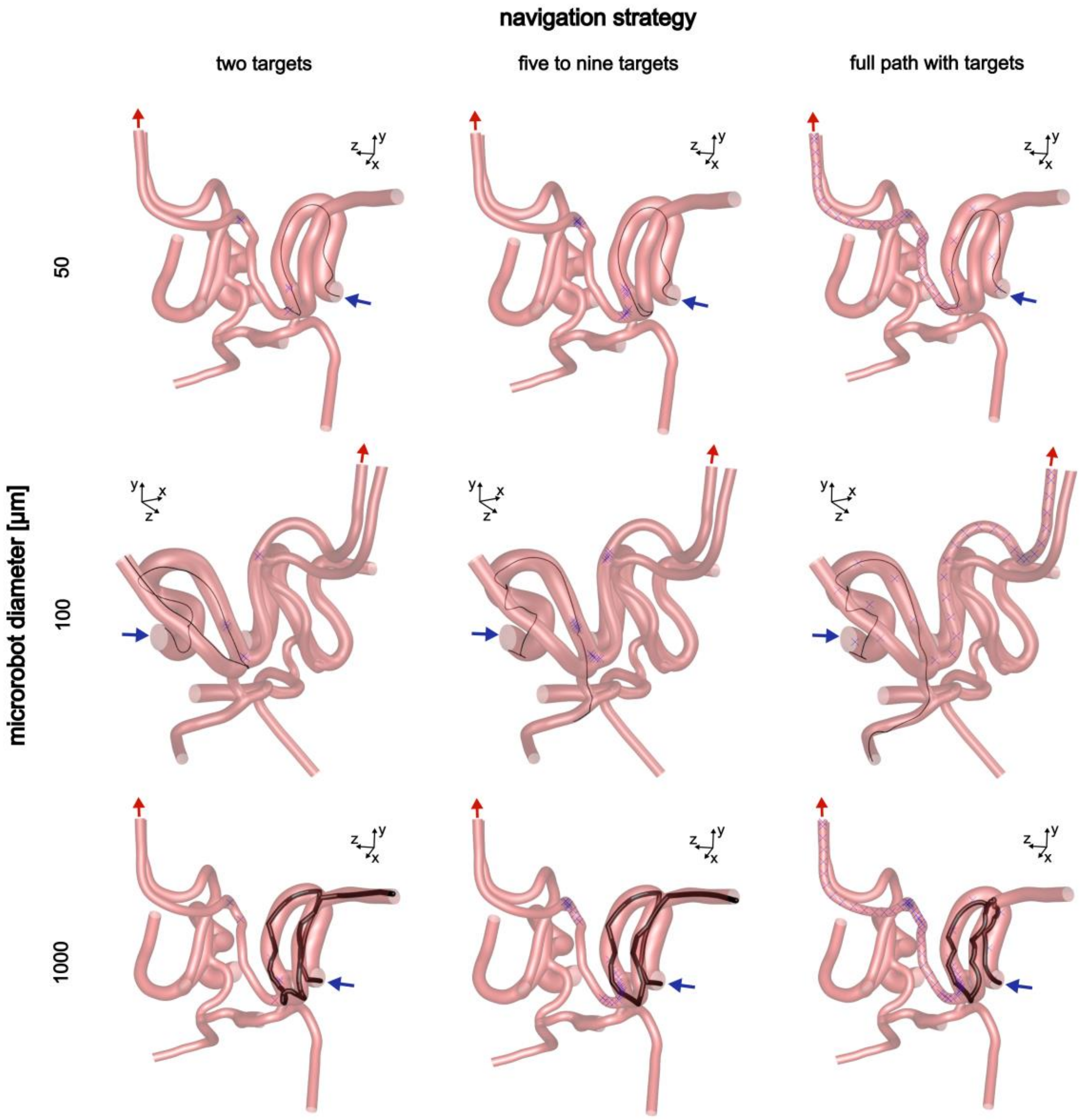

Figure S5 – Example of microrobots of diameter 50, 100 and 1000 µm that did not reach the target vessel due to becoming trapped at the artery walls or being deviated to another outlet. The 50 µm microrobot was navigated from the RICA to RACA using the two targets strategy, with targets placed four microrobot diameters upstream and downstream the position of flow splitting in each bifurcation; using the five to nine targets strategy with five targets, 500 µm apart in each bifurcation; and using the full path with targets strategy with the targets separated by five times the radial distance available for the 1000 µm microrobot to move inside each artery without touching the walls (i.e. 5 × (D_a − 1000 µm) in Table 2). The 100 µm microrobot was navigated from the LICA to the LACA with targets placed three microrobot diameters upstream and downstream the position of flow splitting in each bifurcation; using the five to nine targets strategy with five targets, 500 µm apart in each bifurcation; and using the full path with targets strategy with the targets separated by five times the radial distance available for the 1000 µm microrobot to move inside each artery without touching the walls (i.e. 5 × (D_a − 1000 µm) in Table 2). The 1000 µm microrobot was navigated from the RICA to the RACA with targets placed four microrobot diameters upstream and one microrobot diameter downstream the position of flow splitting in each bifurcation; using the five to nine targets strategy with nine targets, 1000 µm apart in each bifurcation; and using the full path with targets strategy with the targets separated by five times the radial distance available for the 1000 µm microrobot to move inside each artery without touching the walls (i.e. 5 × (D_a − 1000 µm) in Table 2).The inlet and outlet associated with each route are represented by the blue and red arrows, respectively. The microrobots are represented by their spherical volume throughout their trajectory and the intermediate targets are represented by the blue crosses. The microrobots were navigated by imposing the spatially-constant G1 and G2 magnetic gradients.

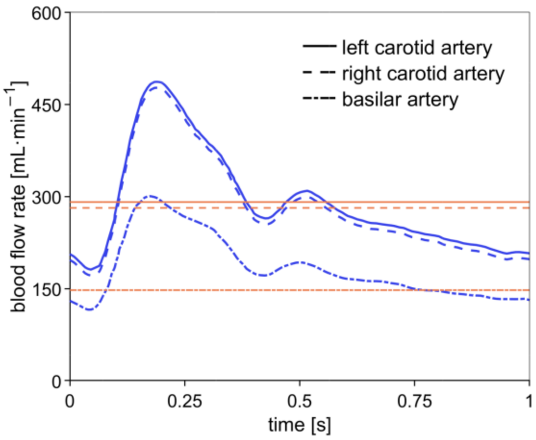

Figure S6 – Typical pulse waves (blue lines) for the carotid and basilar arteries,[13,14] prepared so that the average values (orange lines) matched the flow rates considered in the simulations of sections 3.1 and 3.2.

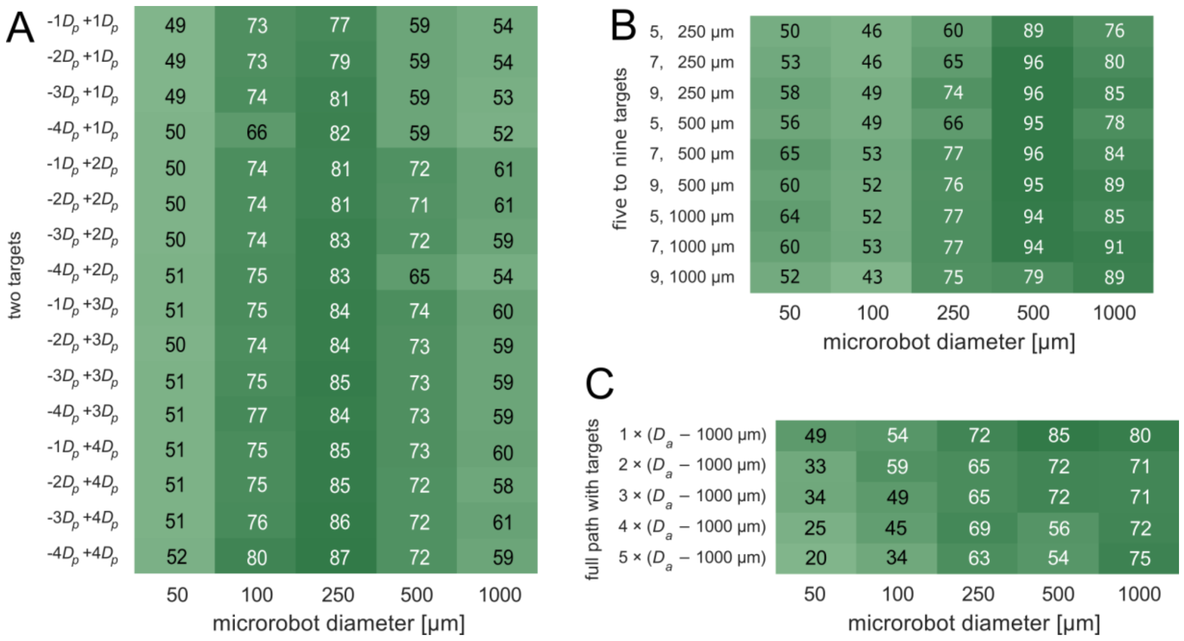

Figure S7 – Navigation success [%] when steering microrobots of five different diameters (50 – 1000 µm) with different number and positioning of the targets in each of the three navigation strategies considered in this work: A – Two targets strategy, B - Five to nine targets strategy, and C – Full path with targets strategy. In this analysis, the flow rate imposed at the inlets corresponds to the maximum observed in the pulse waves of Figure S6. The navigation success was calculated as the ratio between the number of microrobots reaching the target vessels and the number of microrobots released at the inlets (i.e. at the 279 – 1754 different entrance positions considered, which depend on the inlet and microrobot diameters, Table S1). In each table cell, n = 9024, 8236, 5196, 5236 and 2868 for the microrobot sizes of 50, 100, 250, 500 and 1000 µm, respectively

A — two targets (navigation success [%], microrobot diameter [µm])

two targets	50	100	250	500	1000
$-1D_p$ $+1D_p$	16	38	54	41	62
$-2D_p$ $+1D_p$	16	40	54	43	62
$-3D_p$ $+1D_p$	17	40	54	52	58
$-4D_p$ $+1D_p$	18	48	54	45	54
$-1D_p$ $+2D_p$	16	38	54	42	80
$-2D_p$ $+2D_p$	17	41	54	58	69
$-3D_p$ $+2D_p$	17	42	54	56	70
$-4D_p$ $+2D_p$	18	47	54	50	63
$-1D_p$ $+3D_p$	17	40	54	53	84
$-2D_p$ $+3D_p$	17	41	54	63	69
$-3D_p$ $+3D_p$	18	42	58	62	68
$-4D_p$ $+3D_p$	18	47	55	56	79
$-1D_p$ $+4D_p$	17	41	54	61	69
$-2D_p$ $+4D_p$	17	42	54	63	69
$-3D_p$ $+4D_p$	18	42	62	63	69
$-4D_p$ $+4D_p$	18	48	55	50	69

B — five to nine targets

five to nine targets	50	100	250	500	1000
5, 250 µm	60	68	62	61	62
7, 250 µm	63	77	69	63	68
9, 250 µm	78	78	79	63	81
5, 500 µm	65	74	67	63	76
7, 500 µm	78	83	80	61	81
9, 500 µm	74	73	80	63	82
5, 1000 µm	73	79	78	61	76
7, 1000 µm	73	71	80	62	82
9, 1000 µm	56	53	57	63	55

C — full path with targets

full path with targets	50	100	250	500	1000
1 × (D_a − 1000 µm)	37	43	74	83	76
2 × (D_a − 1000 µm)	31	32	76	81	77
3 × (D_a − 1000 µm)	16	17	61	78	70
4 × (D_a − 1000 µm)	12	14	30	55	65
5 × (D_a − 1000 µm)	15	19	22	19	30

Figure S8 - Navigation success [%] when steering microrobots of five different diameters (50 – 1000 µm) with different number and positioning of the targets in each of the three navigation strategies considered in this work: A – Two targets strategy, B - Five to nine targets strategy, and C – Full path with targets strategy. In this analysis, the flow rate imposed at the inlets corresponds to the minimum observed in the pulse waves of Figure S6. The navigation success was calculated as the ratio between the number of microrobots reaching the target vessels and the number of microrobots released at the inlets (i.e. at the 279 – 1754 different entrance positions considered, which depend on the inlet and microrobot diameters, Table S1). In each table cell, n = 9024, 8236, 5196, 5236 and 2868 for the microrobot sizes of 50, 100, 250, 500 and 1000 µm, respectively

A — two targets (microrobot diameter [µm])

two targets	50	100	250	500	1000
$-1D_p$ $+1D_p$	2	3	12	33	12
$-2D_p$ $+1D_p$	2	3	1	33	5
$-3D_p$ $+1D_p$	2	3	3	19	6
$-4D_p$ $+1D_p$	2	4	4	26	4
$-1D_p$ $+2D_p$	1	4	11	32	28
$-2D_p$ $+2D_p$	1	3	2	19	21
$-3D_p$ $+2D_p$	1	1	3	21	32
$-4D_p$ $+2D_p$	1	8	3	26	19
$-1D_p$ $+3D_p$	1	3	13	27	16
$-2D_p$ $+3D_p$	1	3	8	21	27
$-3D_p$ $+3D_p$	1	1	7	25	30
$-4D_p$ $+3D_p$	1	8	4	26	21
$-1D_p$ $+4D_p$	1	2	21	21	22
$-2D_p$ $+4D_p$	1	3	8	21	22
$-3D_p$ $+4D_p$	1	2	4	24	31
$-4D_p$ $+4D_p$	0	10	5	29	28

B — five to nine targets

five to nine targets	50	100	250	500	1000
5, 250 µm	5	3	12	15	15
7, 250 µm	1	2	17	13	14
9, 250 µm	3	4	13	13	7
5, 500 µm	1	0	16	13	12
7, 500 µm	7	5	11	13	11
9, 500 µm	3	10	11	14	10
5, 1000 µm	1	3	7	14	13
7, 1000 µm	4	11	9	15	11
9, 1000 µm	4	6	13	11	4

C — full path with targets

full path with targets	50	100	250	500	1000
1 × (D_a − 1000 µm)	10	5	2	1	0
2 × (D_a − 1000 µm)	4	1	9	2	7
3 × (D_a − 1000 µm)	5	4	13	2	12
4 × (D_a − 1000 µm)	4	3	20	19	7
5 × (D_a − 1000 µm)	5	7	1	11	3

Figure S9 – Difference between the navigation success [%] obtained with the flow rates of Table 3 and the average navigation success when using the maximum and minimum flow rates observed in the pulse waves of Figure S6. The navigation success in each of the abovementioned cases was obtained when steering microrobots of five different diameters (50 – 1000 µm) with different number and positioning of the targets in each of the three navigation strategies considered in this work: A – Two targets strategy, B - Five to nine targets strategy, and C – Full path with targets strategy.

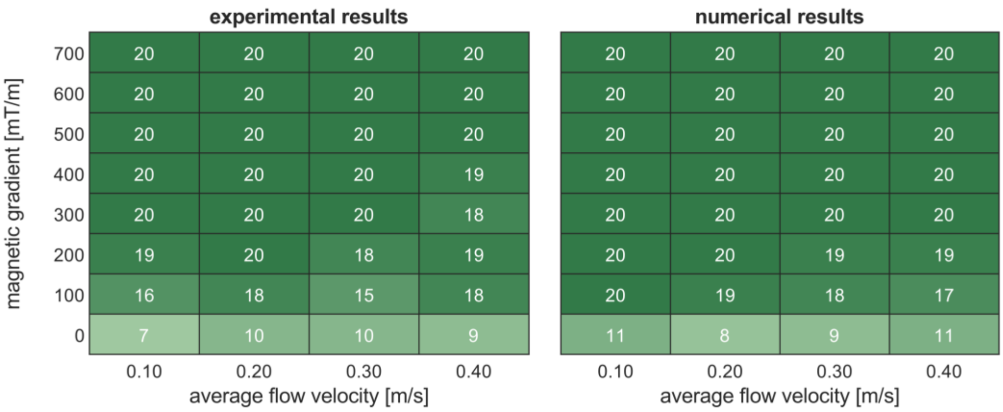

Figure S10 – Number of spheres that reached the desired bifurcation outlet (out of 20 that entered the bifurcation) for the *in vitro* experiments and for the numerical simulations. The presented results were obtained considering the different conditions of flow velocity and magnetic gradient imposed and show that the proposed modeling approach can adequately predict the navigation success observed in the *in vitro* experiments. Minor differences can be seen for the lower magnetic gradients considered (i.e. 0 to 400 mT m^{-1}), for which the number of spheres predicted to reach the desired bifurcation outlet in the simulations and that observed in the experiments differed by 0–4 out of the 20 released in each experimental condition. Such differences may be due to a slight mismatch in the spheres entrance position, velocity magnitude and direction, and sphere-wall coefficient of restitution, and those prevailing in the experimental setup, resulting in slightly different trajectories along the bifurcation and diversion of some spheres to the other outlet.

References

1. Fukushima, T., Homma, T., Azuma, T. & Harakawa, K. Characteristics of secondary flow in steady and pulsatile flows through a symmetrical bifurcation. *Biorheology* **24**, 3–12 (1987).

2. Karmonik, C. *et al.* Quantitative comparison of hemodynamic parameters from steady and transient CFD simulations in cerebral aneurysms with focus on the aneurysm ostium. *J Neurointerv Surg* **7**, 367–372 (2015).

3. Mikhal, J., H., C. & J., B. Simulation of Pulsatile Flow in Cerebral Aneurysms: From Medical Images to Flow and Forces. *Aneurysm* (2012) doi:10.5772/47858.

4. Kim, C. S., Kiris, C., Kwak, D. & David, T. Numerical simulation of local blood flow in the carotid and cerebral arteries under altered gravity. *J Biomech Eng* **128**, 194–202 (2006).

5. Malcolm, A. D. & Roach, M. R. Flow disturbances at the apex and lateral angles of a variety of bifurcation models and their role in development and manifestations of arterial disease. *Stroke* **10**, 335–343 (1979).

6. Ku, D. N., Giddens, D. P., Zarins, C. K. & Glagov, S. Pulsatile flow and atherosclerosis in the human carotid bifurcation. Positive correlation between plaque location and low and oscillating shear stress. *Arteriosclerosis* **5**, 293–302 (1985).

7. Chen, L. Hemodynamics in the cerebral circulation : numerical studies and experimental investigation. (2005).

8. Hillen, B., Drinkenburg, B. A. H., Hoogstraten, H. W. & Post, L. Analysis of flow and vascular resistance in a model of the cricle of Willis. *J Biomech* **21**, 807–814 (1988).

9. Fahy, P. *et al.* An experimental investigation of the hemodynamic variations due to aplastic vessels within three-dimensional phantom models of the circle of willis. *Ann Biomed Eng* **42**, 123–138 (2014).

10. Rabby, M. G., Shupti, S. P. & Molla, Md. M. Pulsatile Non-Newtonian Laminar Blood Flows through Arterial Double Stenoses. *Journal of Fluids* **2014**, 1–13 (2014).

11. Berselli, L. C., Miloro, P., Menciassi, A. & Sinibaldi, E. Exact solution to the inverse Womersley problem for pulsatile flows in cylindrical vessels, with application to magnetic particle targeting. *Appl Math Comput* **219**, 5717–5729 (2013).

12. Bushi, D. *et al.* Hemodynamic evaluation of embolic trajectory in an arterial bifurcation: An in-vitro experimental model. *Stroke* **36**, 2696–2700 (2005).

13. Manini, S., Antiga, L., Botti, L. & Remuzzi, A. pyNS: An Open-Source Framework for 0D Haemodynamic Modelling. *Ann Biomed Eng* **43**, 1461–1473 (2015).

14. Xiang, J., Siddiqui, A. H. & Meng, H. The effect of inlet waveforms on computational hemodynamics of patient-specific intracranial aneurysms. *J Biomech* **47**, 3882–3890 (2014).

15. Enzmann, D. R., Ross, M. R., Marks, M. P. & Pelc, N. J. Blood flow in major cerebral arteries measured by phase-contrast cine MR. *AJNR Am J Neuroradiol* **15**, 123–9 (1994).

16. Bammer, R., Hope, T. A., Aksoy, M. & Alley, M. T. Time-resolved 3D quantitative flow MRI of the major intracranial vessels: Initial experience and comparative evaluation at 1.5T and 3.0T in combination with parallel imaging. *Magn Reson Med* **57**, 127–140 (2007).

17. Zhao, M. *et al.* Regional Cerebral Blood Flow Using Quantitative MR Angiography. *American Journal of Neuroradiology* **28**, 1470–1473 (2007).

18. Tanaka, H. *et al.* Relationship between variations in the circle of Willis and flow rates in internal carotid and basilar arteries determined by means of magnetic resonance imaging with semiautomated lumen segmentation: reference data from 125 healthy volunteers. *AJNR Am J Neuroradiol* **27**, 1770–5 (2006).

19. Haverkort, J. W., Kenjereš, S. & Kleijn, C. R. Computational simulations of magnetic particle capture in arterial flows. *Ann Biomed Eng* **37**, 2436–2448 (2009).

20. Upton, G. J. G. & Jobson, J. D. *Applied Multivariate Data Analysis, Volume 1: Regression and Experimental Design*. *The Mathematical Gazette* vol. 78 (1994).
